# Auditory Hair Cell Mechanotransduction Channels Dynamically Shape the Mechanical Properties of Their Membrane Environment

**DOI:** 10.1002/advs.202508268

**Published:** 2025-09-04

**Authors:** Shefin S. George, Anthony J. Ricci

**Affiliations:** ^1^ Department of Otolaryngology‐Head and Neck Surgery Stanford University Palo Alto 94304 USA; ^2^ Department of Molecular and Cellular Physiology Stanford University Palo Alto 94304 USA

**Keywords:** auditory, cochlea, hair cell, mechanotransduction, membrane, scramblase, TMC

## Abstract

The plasma membrane is actively regulated by lipid transporters that create electrochemical gradients between leaflets, and passively by scramblases that dissipate these gradients. Membrane properties such as lipid packing are critical for the proper function of transmembrane proteins, particularly mechanosensitive ion channels. Mechanosensation is a key component of many sensory processes including balance, and hearing. Inner ear hair cells convert mechanical deflection of their hair bundles into electrical signals by gating mechanoelectrical transduction (MET) channels. Transmembrane channel‐like proteins (TMCs) are an essential component of the hair cell MET complex, and part of a superfamily of molecules whose members are ion channels and/or lipid scramblases. TMCs are implicated as scramblases in hair cells, however no direct evidence separates scramblase activity from channel properties, nor is there clarity around how MET activity impacts the stereocilia environment. Here, using a novel viscosity sensor boron‐dipyrromethene (BODIPY) 1c, this work probes stereocilia membrane viscosity and its relationship with TMC expression and MET current. Using developmental, genetic, electrophysiological and pharmacological tools, this work demonstrates that the MET complex directly regulates the stereocilia membrane viscosity. This work shows that phosphatidylserine externalization does not completely describe, nor solely represent TMC scramblase activity. Lipid flippase/floppase activity along with an MET independent scramblase are implicated in lipid remodeling. Together these data identify a dynamic regulation of stereocilia membrane hypothesized to modulate mechanotransduction channel properties.

## Graphical abstract

This work shows, for the first time, that the stereocilia membrane in cochlear hair cells is dynamically regulated by the mechanotransduction channel to impact the membrane mechanical properties. This work provides direct evidence that the opening and closing associated with the MET channel is regulating the membrane viscosity suggesting that the MET channel are mechanosensitive membrane scramblases that regulate the stereocilia membrane.

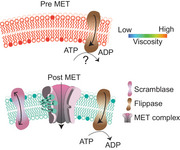

## 1 Introduction

Far from being a passive envelope for cells, biological membranes form active environments that regulate the function of membrane‐associated proteins.^[^ ^1^ ^]^ Biological membranes are asymmetrical, with amine‐containing phospholipids such as phosphatidylserine (PS) and phosphatidylethanolamine (PE) on the inner leaflet, while the choline‐containing lipids, phosphatidylcholine (PC) and sphingomyelin (SM), are on the outer leaflet.^[^ ^2^, ^3^ ^]^ This asymmetrical distribution of phospholipids is maintained by ATP‐dependent lipid transporters, “flippases” and “floppases” while the asymmetry can be disrupted by ATP‐independent lipid “scramblases” that provide permeation pathways for the movement of phospholipids like PS and PE from the inner to the outer leaflet and PC from the outer to the inner leaflet along their concentration gradient.^[^ ^4^ ^]^ Maintenance and loss of membrane phospholipid asymmetry act as signals to facilitate many relevant health and disease related processes such as cell activation and migration, signal transduction, blood coagulation, fertilization, synapse pruning, apoptosis and more.^[^ ^5^, ^6^, ^7^, ^8^ ^]^ Emerging evidence suggests that lipid scrambling reduces lipid packing, thereby impacting membrane mechanical properties like viscosity.^[^ ^3^, ^9^ ^]^ Membrane mechanical properties like curvature, tension, viscosity (or lipid packing efficiency) and thickness are critical for the proper function of a variety of membrane associated proteins, including mechanosensitive ion channels.^[^ ^10^, ^11^ ^]^ Mechanosensation is a key step in many sensory processes, including pain, touch, osmoregulation, proprioception, balance, and hearing. Model systems such as the bacterial MscL channels and the mammalian PIEZO channel have provided important insight into how mechanical strain can impact ion channel activity and the role of the lipid bilayer in modulating this function.^[^ ^10^, ^11^, ^12^, ^13^ ^]^Because the mechanotransduction channel of cochlear hair cells is coupled to the tip link, it is commonly thought that mechanical force regulates channel activity by mechanisms different from those employed by non‐tethered channels that are directly regulated by membrane stretch. However, given the number of transmembrane domains involved in the hair cell mechanotransduction process, it is unlikely that lipid mechanics do not impact force translation and gating energy to some degree.^[^ ^14^ ^]^ Hair cells use an apically located specialized organelle, the hair bundle, to convert mechanical vibrations into electrical activity. Hair bundles consist of an array of actin‐filled membrane protrusions, stereocilia, organized in a staircase pattern. Deflection regulates force on a filament (tip‐link) that connects the shorter stereocilium to its next taller neighbor^[^ ^15^ ^]^ and opens and closes MET channels that reside near the tips of the shorter stereocilia. The tip‐link is connected to multiple elements of the MET machinery, each of which have transmembrane domains.

The stereocilia membrane expresses lipid transporters like ATP8B1 and ATP8A2, lipid binding proteins such as StarD10, PITPNA, PITPNB and OSBPL2 and has specialized lipid domains.^[^ ^16^, ^17^, ^18^ ^]^ The stereocilia membrane is more diffusive than the cell body.^[^ ^19^, ^20^ ^]^ There is growing evidence supporting the hypothesis that hair cell MET is modulated by the stereocilia membrane. For example, divalent cations regulate the MET channel resting open probability (*P*_o_) consistent with a sensitivity to lipid packing.^[^ ^21^ ^]^ GsMTX4, modulates mechanically gated channels by intercalating into the membrane near the channels and raising the energy requirement for channel gating. GsMTX4 shifts the hair cell MET activation (current‐displacement) curves rightward,^[^ ^21^ ^]^ similar to its effect on stretch‐activated channels.^[^ ^22^, ^23^ ^]^ Computational modeling further implicated the bilayer as potentially contributing to adaptation, channel co‐operativity and gating compliance.^[^ ^24^, ^25^, ^26^, ^27^, ^28^ ^]^ The first direct evidence for membrane modulation of MET came from our measurements of membrane diffusivity demonstrating that stereocilia membrane diffusivity is sensitive to calcium and voltage.^[^ ^20^ ^]^ Collectively, these data suggest the stereocilia membrane properties are not simply passive and that they can impact MET channel function.

The key components of the MET channel complex are the transmembrane channel‐like proteins 1 and 2 (TMC1 and TMC2), LHFPL5 and TMIE (transmembrane inner ear).^[^ ^29^, ^30^, ^31^, ^32^, ^33^ ^]^ TMC molecules are putative pore forming subunits that are part of a broader family of proteins including the TMEM16 and OSCA/TMEM63 molecules which can be ion channels and/or lipid transporters, specifically membrane scramblases.^[^ ^34^ ^]^ Initially, externalization of PS, which is usually restricted to the inner leaflet, triggered by MET channel inhibition suggested TMC1 but not TMC2 had calcium inhibited scramblase activity.^[^ ^35^ ^]^ While there is no direct evidence that TMCs are scramblases, there is an accumulating body of indirect evidence including, studies of TMC mutant mice, using PS externalization, supporting that both TMC1 and TMC2 show scramblase activity.^[^ ^36^ ^]^ Apoptosis (a signature of which is PS externalization) has been argued to be triggered indirectly by TMC mutations leading to a reduction in Ca^2+^‐ATPase levels.^[^ ^37^ ^]^ To date, how scramblase activity might regulate local lipid environment and mechanotransduction is missing.

Here, we monitored membrane viscosity using the BODIPY 1c sensor using fluorescence lifetime imaging (FLIM), and live cell membrane scramblase activity using PS‐specific binding of Annexin V (AnV) to directly assess how TMCs might regulate stereocilia membrane properties. Together with developmental, genetic and biophysical tools, we show for the first time that 1) the stereocilia membrane is dynamically regulated to impact the membrane mechanical properties 2) the hair cell MET channel is an integral component of this regulation 3) the opening and closing associated with the MET channel is regulating the effective viscosity suggesting that TMCs are membrane scramblases that regulate the stereocilia membrane in an activity dependent way and 4) PS externalization does not fully represent TMC or scramblase activity, nor does it simply reflect apoptosis. Together these data suggest that TMCs are mechanosensitive scramblases and are part of an active process that regulates membrane mechanics. This work provides a foundation upon which to re‐evaluate MET channel gating and processes related to adaptation, gating springs and potentially cochlear amplification.

## 2 Results

### 2.1 Stereocilia Membrane is Less Viscous Compared to Supporting Cells

**Figure 1:**
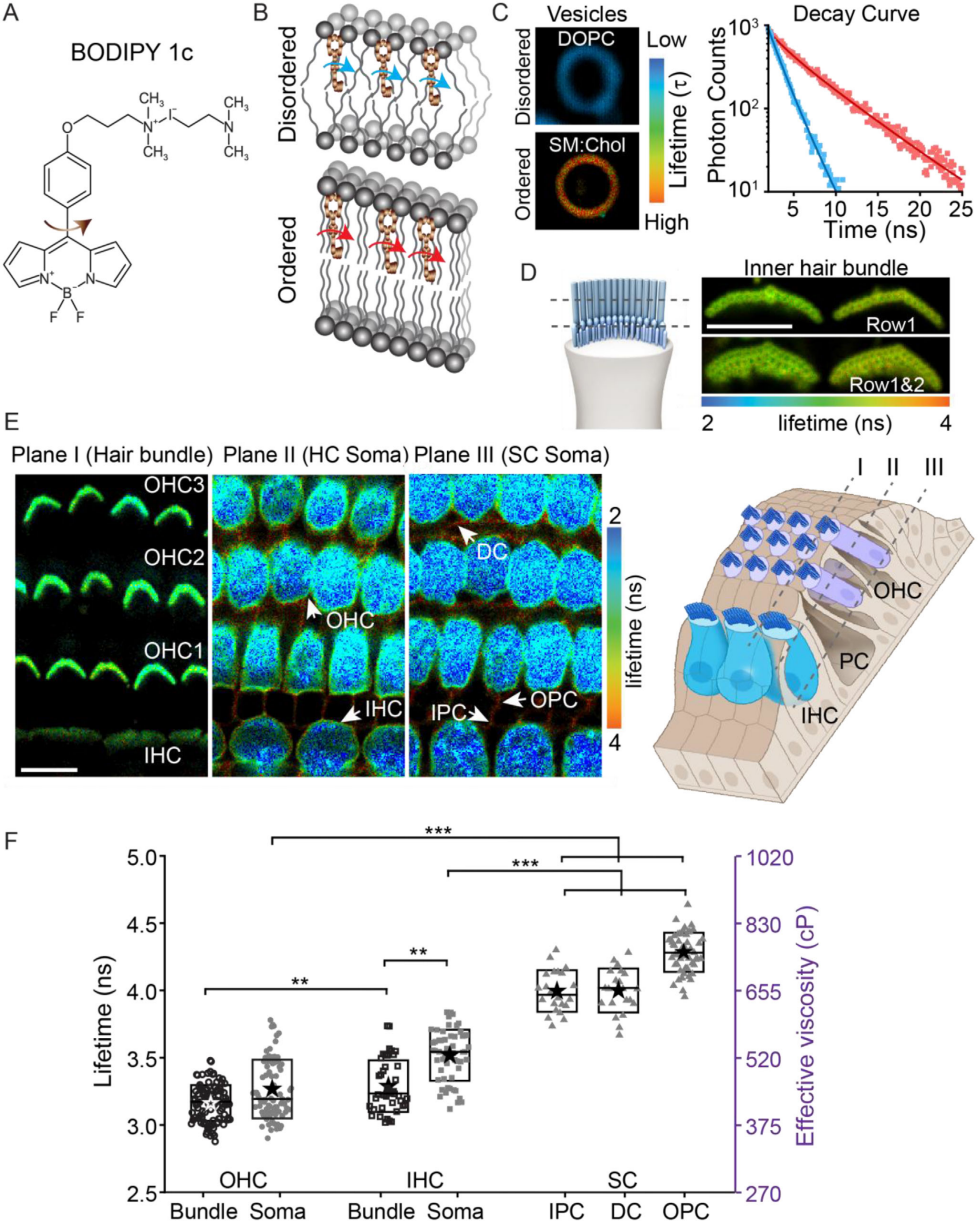
Stereocilia membrane is less viscous compared to pillar cells. A) Molecular structure of BODIPY 1c. B) Schematic showing the relative orientation of BODIPY 1c in an ordered and disordered bilayer, adapted from.^[^ ^39^ ^]^ C) Example FLIM images and decay curves of BODIPY 1c from DOPC and sphingomyelin: cholesterol (SM:Chol) vesicles with low viscosity (blue symbols) and high viscosity (red symbols) and the corresponding exponential fits (solid lines) and example lifetime images generated with each pixel represented by the time constant (τ, mean lifetime) derived from fitting the decay curve from each pixel with a double exponential decay model. D) FLIM images of the inner hair cell (IHC) bundles of P10 rat mid‐apical cochlear turn stained with BODIPY 1c with laser focused on the tops of the first and second row stereocilia with the schematic on the left showing the approximate location of the focal planes. E) Cross‐sectional FLIM images of P10 rat mid‐apical turn stained with BODIPY 1c in XY planes to present sensory cells (IHCs and outer hair cells (OHCs)) and supporting cells (inner pillar cells (IPCs), outer pillar cells (OPCs) and deiter cells (DCs)). Schematic on the right panel illustrates the approximate position of the focal plane of the FLIM images shown in E with grey dashed lines. F) Quantification of mean lifetime (left axis) and effective viscosity (right axis) from different cochlear structures in P10 rat mid‐apical turn (n = 6 animals). Boxes represent the SD, and the star symbol indicates the mean. Each data point corresponds to a hair bundle or a cell (for soma). **p < 0.01, ***p < 0.001. Scale bar = 10 µm.

To measure the cochlear cell's membrane properties, we used a viscosity‐sensitive “molecular rotor” BODIPY 1c (**Figure** **1**A) coupled with fluorescence lifetime imaging (FLIM). Molecular rotors are small synthetic fluorophores for which the fluorescence quantum yield and fluorescence lifetime, that is, the average time a fluorophore remains in the excited state, increase with increases in the viscosity of their immediate environment (Figure 1B).^[^ ^38^, ^39^, ^40^, ^41^ ^]^ To obtain concentration‐independent measurements of viscosity, fluorescence lifetime of BODIPY 1c was used. More details on lifetime measurements, validation and calibration (lifetime versus viscosity plot) of BODIPY 1c and determination of optimal incubation concentration of BODIPY 1c in cochlear hair bundles are presented in the Experimental Section and Figures S1 and S2, Supporting Information. Briefly, to validate the BODIPY 1c rotor under conditions known to exhibit viscosity differences, FLIM was carried out in artificial vesicles of known lipid composition. Liposomes created with pure 1,2‐ioleoyl‐sn‐glycero‐3‐phosphocholine (DOPC) resulting in disordered or less viscous membrane (≈113 cP) generated shorter (blue) decay curve and lifetime while sphingomyelin/cholesterol (SM:Chol) vesicles resulting in more viscous membranes (≈920 cP) gave longer (red) decay curve and lifetime (Figure 1C and Figure S1, Supporting Information). Our control data suggest the sensor reports effective membrane viscosity even though it is not possible to calibrate the sensor in our target system where uncontrolled variables like membrane proteins and cytoskeletal connections could alter the absolute value of the measured viscosity. Data are presented as “effective” viscosity (see dye calibration in Figure S1A–C, Supporting Information) unless otherwise stated. It should be noted that sensor concentration was selected so that the amount of sensor in the membrane was not impacting the effective viscosity measurement. The effective viscosity measurement does not vary between 1 and 10 µM sensor (Figure S2, Supporting Information). The benefit of using lifetime is that lifetime is independent of concentration and simply requires the appropriate number of photons to be counted to define the curve (see details in supplementary material).

Example FLIM images from live postnatal day 10 (P10) rat mid‐apical (2–3 kHz)^[^ ^42^ ^]^ inner hair cell bundles stained with BODIPY 1c at the locations indicated in the schematic, ostensibly the tops of the first row and second row stereocilia, show the donut‐like fluorescent rings attesting to stereocilia membrane localization of the BODIPY 1c (Figure 1D). To investigate the viscosity differences between different structures of the organ of Corti, especially between the hair bundles and soma and between the sensory cells and the supporting cells, FLIM z‐stacks of the organ of Corti were acquired. Figure 1E shows cross‐sectional FLIM images of P10 rat mid‐apical turn stained with BODIPY 1c in XY planes (illustrated on the right panel with grey dashed lines) to present sensory cells – inner hair cells (IHCs) and outer hair cells (OHCs) and supporting cells – inner pillar cells (IPCs), outer pillar cells (OPCs) and Deiters’ cells (DCs). The fluorescence decay of BODIPY 1c in most cochlear structures, including hair bundles and soma of HCs and supporting cells, at 20 °C was best fitted with a biexponential model indicating that the rotor is probing the heterogeneities in the plasma membrane.^[^ ^38^, ^43^ ^]^ Using the time constants extracted from the biexponential decay, we report the intensity‐weighted mean lifetime to represent the average lifetime. The measured lifetime was significantly longer, and the effective viscosity was higher for IHC bundles (456 ± 50 cP) than that of the OHC bundles (404 ± 25 cP; *t*‐test, *p < 0.01*), and the effective viscosity of the sensory cells (IHCs and OHCs) bundles and soma (IHC soma at 530 ± 30 cP and OHC soma at 449 ± 45 cP) were significantly lower than that of the supporting cells – IPCs (653 ± 45 cP), OPCs (764 ± 55 cP) and DCs (654 ± 47 cP) (*t*‐test, *p < 0.001*) (Figure 1F). For the IHCs, the bundles had significantly shorter lifetime and thus lower effective viscosity than that of the soma (*t*‐test, *p < 0.01*). For reference, the viscosity values we observed for the pure DOPC vesicle was ≈110 cP and for the SM:Chol (70:30) vesicle was ≈920 cP (Figure S1D–F, Supporting Information).

Given that BODIPY 1c was found in the cytoplasmic membranes of HCs and not supporting cells, we examined whether the dye could enter the HCs through MET channels by monitoring fluorescence intensity in the soma treated with well‐characterized MET channel blockers, curare and amiloride.^[^ ^44^, ^45^, ^46^, ^47^, ^48^ ^]^ Incubation with 1 mM curare or amiloride resulted in a significant reduction of intensity in the soma of HCs and not PCs (Figure S3A–D, Supporting Information, *t*‐test, *p < 0.001*), indicating that BODIPY 1c enters hair cells at least in part through MET channels; a useful property we exploited to identify hair cells with open MET channels. The cytoplasmic BODIPY 1c fluorescence also correlated with the cytoplasmic signal of FM 4–64, (Figure S3E,F, Supporting Information; *r^2^* = 0.79) a fluorescent dye previously shown to enter hair cells via MET channels.^[^ ^49^, ^50^, ^51^ ^]^ Because HC soma are filled with intracellular membranes and the sensor entered the soma, the soma membrane lifetime measurements reported in Figure 1F likely represent a weighted average that includes some of the intracellular membranes. This error could underestimate the lifetime of the HC soma membrane. For this reason, we did not pursue HC soma comparisons further. Supporting cells did not take up the sensor at any age and the effective viscosity of supporting cells was high and constant over the time course and between the different mice variants used in these studies.

### 2.2 Membrane Viscosity is Significantly High in Mice Lacking MET Channel Complex

We first explored whether the lack of MET channel complex in the hair bundle would affect the stereocilia membrane effective viscosity. For this purpose, we first captured FLIM images of organ of Corti of the homozygous spinner *Tmie^−/−^* mice^[^ ^52^ ^]^ which lack TMIE, a subunit contributing to the channel pore and required for localization of other putative pore forming subunits TMC1 and TMC2 to the hair bundle.^[^ ^29^, ^50^, ^53^ ^]^ We also investigated the membrane viscosity in *Tmc1* and *Tmc2* double knockout mice (*Tmc1* ^−/−^; *Tmc2* ^−/−^), which lack functional MET channels.^[^ ^30^, ^54^, ^55^, ^56^ ^]^

**Figure 2:**
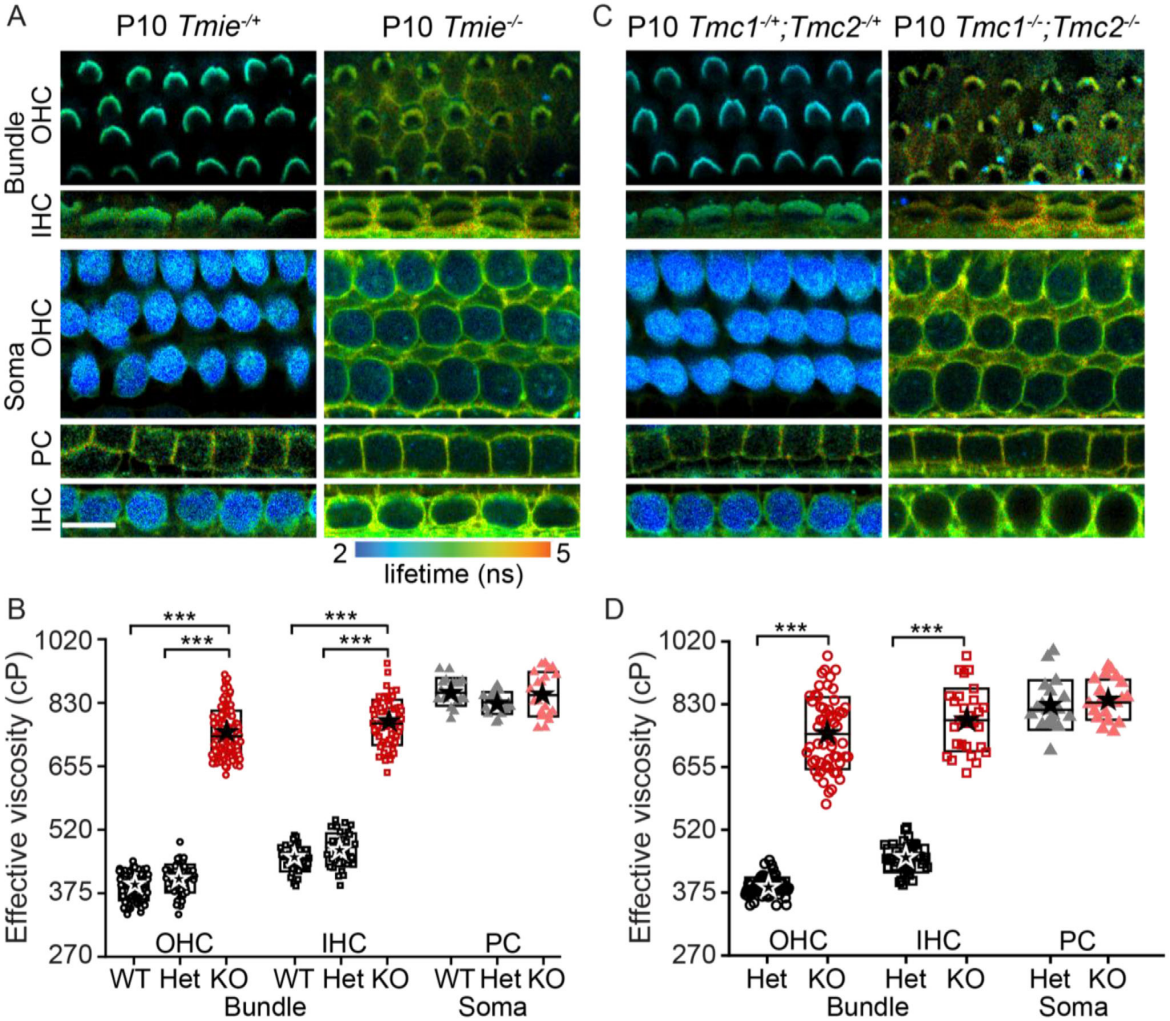
Membrane viscosity is significantly high in mice lacking MET channel. A) FLIM images of P10 mid‐apical *Tmie* ^−/−^ and *Tmie* ^−/+^ hair bundles (top rows) and soma (bottom rows). B) Quantification of the effective viscosity from mutants (n = 8 animals), littermate controls (n = 8 animals) and wild‐type mice (n = 8 animals). C) FLIM images of P10 mid‐apical *Tmc1* ^−/−^;*Tmc2* ^−/−^ and *Tmc1* ^−/+^;*Tmc2* ^−/+^ HC bundles (top rows) and soma (bottom row). D) Quantification of effective viscosity from *Tmc1* ^−/−^;*Tmc2* ^−/−^ (n = 5 animals)and littermate controls (n = 5 animals). Boxes in B and D represent the SD, and the star symbol indicates the mean. Each data point corresponds to a hair bundle or a cell (for soma). *p < 0.05, **p < 0.01, ***p < 0.001. Scale bar = 10 µm.

*Tmie^−/−^* and *Tmc1^−/−;^ Tmc2^−/−^* mice show a loss of all MET current, with both mice resulting in a loss of TMC molecules in the stereocilia.^[^ ^29^, ^57^ ^]^ Both in *Tmie^−/−^* and *Tmc1^−/‐;^ Tmc2^−/−^* mice, the hair bundles in the OHCs and the IHCs showed longer lifetimes (**Figure** **2**A,C; first and second row panels) indicating significantly higher effective viscosity (763 ± 45 cP for OHC and 790 ± 55 cP for IHC for *Tmie^−/−^* and 745 ± 92 cP for OHC and 795 ± 85 cP for IHC for *Tmc1^−/‐;^ Tmc2^−/−^*; Figure 2B,D; *t*‐test, *p<0.001*) compared to their littermate controls, *Tmie^−/+^* (402 ± 35 cP for OHC and 480 ± 40 cP for IHC) and *Tmc1^−/+;^Tmc2^−/+^* (385 ± 25 cP for OHC and 452 ± 48 cP for IHC) and WT (400 ± 35 cP for OHC and 451 ± 28 cP) mice. The images focused on the soma of the HCs (Figure 2A,C; bottom row panels) showed the absence of BODIPY 1c in the cytoplasm of the mutants compared to the littermate controls, confirming that the dye couldn't enter the soma of hair cells without functional MET channels. No significant difference was observed between the pillar cell (PC) of the mutants (840–870 cP) and the littermate controls and WT mice (830–870 cP) suggesting that the effect on the membrane viscosity is specific to the HCs (Figure 2B,D). The prominence of the fluorescence from the supporting cells in the mutants is a consequence of the longer imaging time required to collect sufficient photons to monitor lifetime in the higher effective viscous conditions (as less dye is taken up). Also, the longer imaging times in the control mice would have saturated the hair cell cytoplasm signal contributing to the differences in images. Overall, the data from mice lacking TMC1 and TMC2 demonstrates that either TMC molecules or a functional MET channel complex is required for reducing membrane viscosity in mammalian cochlear hair bundles.

### 2.3 Stereocilia Membrane Viscosity Decreases during Development

**Figure 3:**
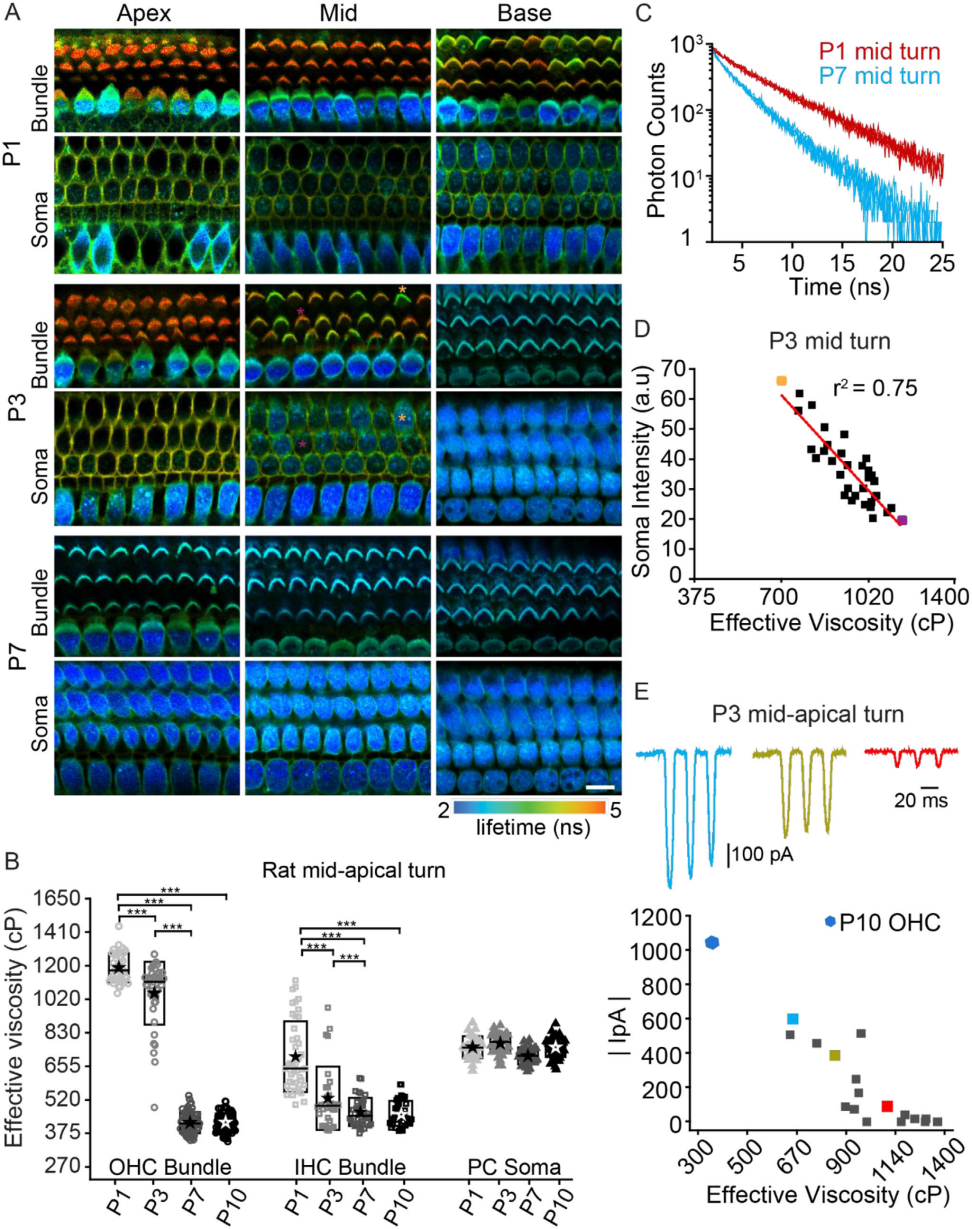
Stereocilia membrane viscosity decreases during development and correlates with the onset of MET. A) FLIM images of hair bundles and soma of developing rat organ of Corti stained with BODIPY 1c from three positions (apex, middle, base) along the cochlea at ages from P1 to P7. B) Quantification of effective viscosity for mid‐apical OHC bundles (open circle), IHC bundles (open square) and PC soma (closed triangle) at P1 (n = 4 animals), P3 (n = 4 animals), P7 (n = 6 animals) and P10 (n = 7 animals). C) Fluorescence decay from P1 (red traces) and P7 (green traces) hair bundles from middle turn. D) Plot showing the correlation between the fluorescence intensity of BODIPY 1c in the soma and the bundle viscosity. Orange and magenta data points correspond to the cells/bundles marked with orange and magenta asterisk in A. The x‐axis is extended to cover the range of viscosities observed in the hair bundles during the development. E) Plot showing the correlation between the MET current and the hair bundle viscosity measured from the corresponding cell (n = 17 cells). The typical value recorded for P10 OHC is highlighted as blue hexagon. Boxes in (B) represent the SD, and the star symbol indicates the mean and the line indicates the median. Each data point corresponds to a hair bundle or a cell (for soma). ***p < 0.001. Scale bar = 10 µm.

Along with the morphological changes in stereocilia height, width and number of rows per hair bundle, mechanosensitivity matures over early postnatal days (day 0 to day 10) during development with a spatial and temporal progression of both functional and structural maturation of the hair bundle.^[^ ^58^ ^]^ The expression pattern of essential MET components such as TMC1 and TMC2 vary during development, specifically within the cochlea, with TMC2 being expressed early postnatally but then replaced over time by TMC1.^[^ ^33^, ^54^, ^57^, ^59^, ^60^ ^]^ We used this temporal and tonotopic windows to explore whether membrane viscosity was impacted by MET. Three locations along the cochlea, apex (1–2 kHz), middle (≈15 kHz) and base (≈50 kHz), based on,^[^ ^42^ ^]^ were monitored from P1‐P10 rats (**Figure** **3**A,C). Overall, hair bundles that are not mechanically sensitive had longer lifetimes (indicative of higher viscosities). At P1, OHCs at apex and middle regions did not have MET currents and their hair bundles had higher effective viscosity. The lack of MET currents is illustrated by the absence of BODIPY 1c in the hair cell soma. OHCs at the P1 base appeared as a mosaic pattern with hair bundles having a broader range of viscosities. Unexpectedly, IHC hair bundles had lower viscosities in all regions with the apex having a mosaic pattern at P1 while the base bundles had low effective viscosity and filled soma, indicative of open MET channels. Consistent with these findings, IHC soma were more filled at each position suggesting more functional MET channels in IHCs. At P3, OHC hair bundles showed lower viscosities in the base with the mosaic pattern having moved to the mid region and the apex remaining with high viscosity. By P7, OHC hair bundles from all regions had lower viscosities and soma were filled. Figure 3B summarizes the developmental reduction in hair bundle effective viscosity for the mid‐apical (2–3 kHz) turn (*t*‐test, *p < 0.001*). Both IHC (705 ± 175 cP at P1, 523 ± 145 cP at P3, 470 ± 60 cP at P7, 460 ± 62 cP at P10) and OHC (1185 ± 75 cP at P1, 1040 ± 160 cP at P3, 425 ± 50 cP at P7, 430 ± 55 cP at P10) bundles show viscosities that reduce during development, where IHC bundles’ viscosity reduces at least 1 day earlier than OHC bundles. During this same time course, no changes in viscosity were found in PCs (750–830 cP) demonstrating that effective viscosity changes were hair cell selective. These data show that membrane viscosity decreases as MET currents mature.

The relationship between effective viscosity and MET maturation is further illustrated by the strong negative correlation between hair bundle viscosity and hair cell uptake of the sensor (Figure 3D, r^2^ = 0.75, *p* < 0.001) measured from the P3 middle turn region shown in Figure 3A. To directly demonstrate that hair bundle viscosity is correlated with MET maturation, we first captured FLIM images and then used whole‐cell recordings to voltage clamp hair cells and measure maximal MET currents using a fluid jet stimulator.^[^ ^61^ ^]^ P3 mid‐apex was selected as there was a broad range of viscosities for the hair bundles. The plot in Figure 3E demonstrates a strong negative correlation between hair bundle viscosity and MET current (r^2^ = 0.67, *p* < 0.001) and that viscosities above 1000 cP are from hair bundles that have no MET currents.

### 2.4 Sensor Distribution between Leaflets Does Not Drive Observed Viscosity Differences

Inner leaflets of plasma membrane are typically less viscous than outer leaflets due to the membrane composition and likely in response to curvature differences.^[^ ^2^, ^3^ ^]^ Given that the sensor can traverse the MET channel under different conditions, it is possible that cells with open MET channels have more sensor in the inner leaflet than those without MET channels open and the presented differences reflect sensor distribution and not effective viscosity. We tested this hypothesis by extracting the sensor from the outer leaflet using fatty‐acid free bovine serum albumin (BSA)^[^ ^2^, ^3^ ^]^ and comparing effective viscosity before and after extraction as well as comparing the percent of sensor extracted, assuming that dye intensity correlates directly with amount of dye. We used the mosaic viscosity region observed at P3 to investigate leaflet distribution as it allows a broad range of the hair bundle viscosity measurements to be included in each experiment. Figure S4A, Supporting Information shows examples of intensity and lifetime images pre and post sensor extraction with BSA. The images show a large reduction in intensity suggesting there is a large component of sensor in the outer leaflet. Changes in lifetime were much more subtle. The summary data in Figure S4B, Supporting Information shows that faster the lifetime, the lower proportion of sensor was found in the outer leaflet, presumably reflecting dye entry through MET channels. Lifetime changes were minimal, mostly less than 10% and mostly observed in the hair bundles with the lower initial effective viscosity. These results support the idea that the inner leaflet is less viscous than the outer leaflet but the difference is not dramatic. These data also show that the sensor distribution varied from 50% to about 35% in the inner leaflet based on initial effective viscosity. Figure S4C, Supporting Information plots the effective viscosity measurements pre v.s. post extraction to further demonstrate the small change observed and that the change largely only happened for cells with initial low effective viscosity. Overall, these data demonstrate that sensor distribution does not explain the large changes in effective viscosity observed during development. Data also provide further support that the sensor enters MET channels as a major pathway to the inner leaflet.

### 2.5 Membrane Viscosity is Significantly High in Mice Lacking TMC1 or TMC2

**Figure 4:**
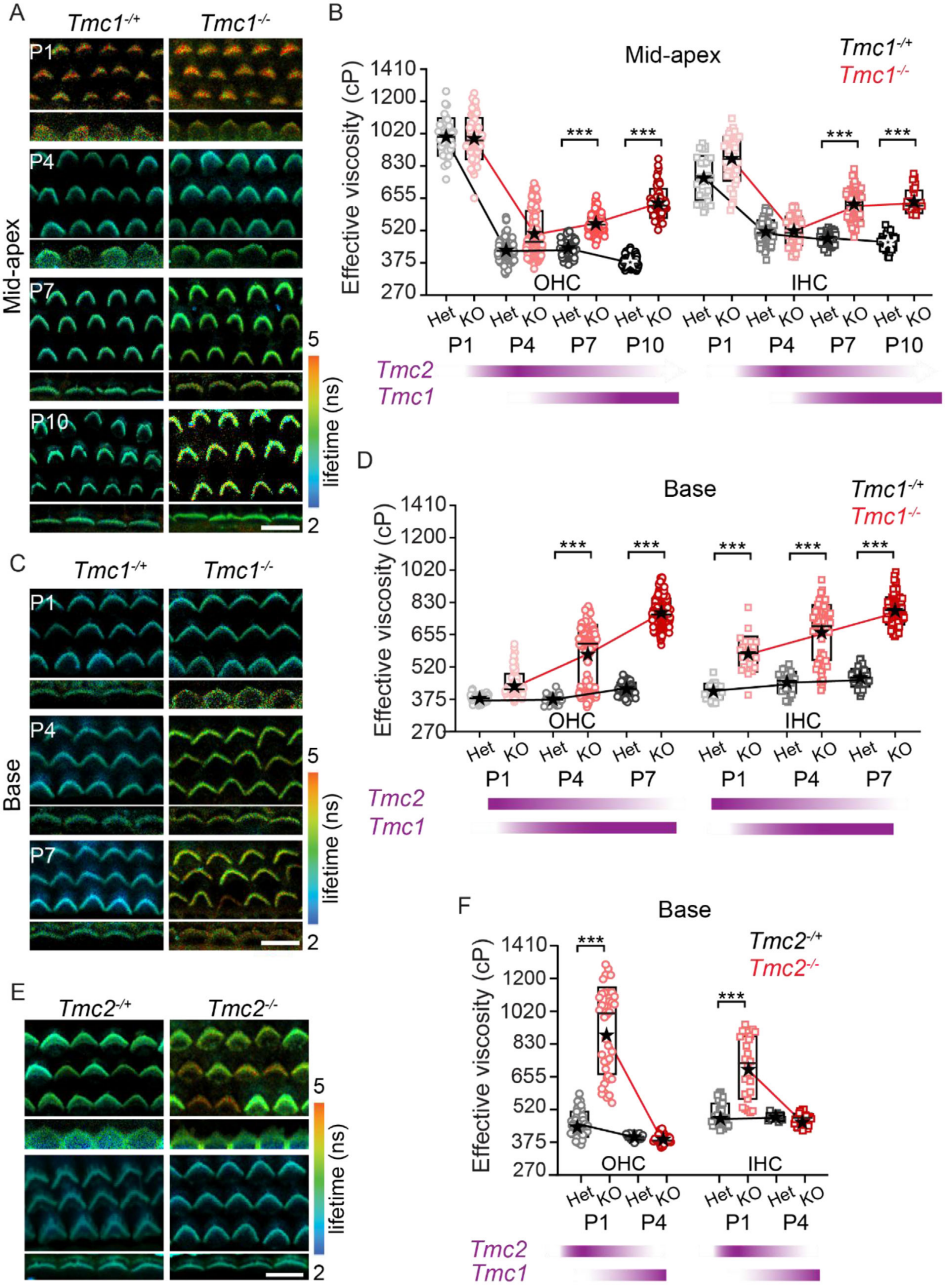
TMC1 and TMC2 are required for the reduction of membrane viscosity during development. FLIM images of OHC and IHC bundles from A) mid‐apical turn of P1, P4, P7 and P10 and, C) basal turn of P1, P4 and P7 mice cochlea from *Tmc1* ^−/−^ and *Tmc1* ^−/+^. The measured hair bundle viscosity for both controls and *Tmc1* ^−/−^ from B) mid‐apical turn (P1 (n = 3 controls and 4 mutants), P4 (n = 6 controls and 6 mutants), P7 (n = 4 controls and 9 mutants) and P10 (n = 8 controls and 6 mutants)) and D) basal turn (P1 (n = 3 controls and 4 mutants), P4 (n = 3 controls and 8 mutants), P7 (n = 5 controls and 10 mutants). E) FLIM images of P1 and P4 basal *Tmc2^−/−^* and *Tmc2^−/+^* mice hair bundles. F) The effective viscosity from the *Tmc2^−/−^* and *Tmc2^−/+^* mice hair bundles at P1 (n = 3 controls and 4 mutants) and P4 (n = 3 controls and 3 mutants). Purple bars at the bottom of B, D and F correspond to the relative expression of TMC1 and TMC2 in the hair cells during the age shown above the bars in corresponding cochlear location. Boxes in B, D and F represent the SD, and the star symbol indicates the mean and the line indicates the median. Each data point corresponds to a hair bundle. ***p < 0.001. Scale bar = 10 µm.

We next wanted to determine if the TMC molecules were directly involved in the changes in membrane viscosity. TMC1 expresses later in time allowing normal development to happen, thus using the *Tmc1^−/−^* separates the protein from development. Two locations along the cochlea, mid‐apex and base, were monitored in P1‐P10 *Tmc1^−/+^* and *Tmc1^−/−^* mice (**Figure** **4**A,C). The hair bundle viscosity appears normal at P4 for mid‐apical turn (420 ± 60 cP for OHC and 505 ± 70 cP for IHB) and at P1 for basal turn (380 ± 35 cP for OHC and 410 ± 40 cP for IHB) where TMC2 is expressed. This demonstrates that TMC2 substitutes for TMC1 in impacting effective viscosity. Viscosity increases in the *Tmc1^−/−^* mice after P4 implicating TMC1 as part of the membrane viscosity regulation. In the mid‐apical turn, MET onset is late,^[^ ^58^ ^]^ and effective viscosity similarly reduces late (Figure 4A,B). The littermate *Tmc1^−/+^* mice maintain lower viscosities after P4 whereas the *Tmc1^−/−^* mice show an increase (*t*‐test, *p < 0.001*) in viscosity for both IHCs (630 ± 60 cP at P7 and 642 ± 55 cP at P10) and OHCs (550 ± 50 cP at P7 and 635 ± 65 cP at P10). Similarly, in the basal region where MET onset happens at or before P1, viscosity of the hair bundles is low and maintained low for littermate controls; in contrast the *Tmc1^−/−^* have an increasing viscosity (580 ± 110 cP for OHC and 660 ± 140 cP for IHC at P4, 740 ± 80 cP for OHC and 780 ± 70 cP for IHB at P7; Figure 4C,D; *t*‐test, *p <0.001*).

It was surprising that viscosity in the *Tmc1^−/−^* did not go as high as pre‐MET onset levels (≈1020 cP at P1 and 640 cP at P10 mid‐apical turn; Figure 4B). We next assessed the hair bundle viscosity and the hair cell soma dye uptake in the corresponding cell in the *Tmc1^−/−^* at P10 mid‐apical region. Inspection of the OHC soma shows a continued uptake of the sensor indicating continued MET current (Figure S5A, Supporting Information; bottom panel). Hereto plotting the bundle viscosity against soma intensity provides a strong correlation (r^2^ = 0.84 in the example provided, Figure S5B, Supporting Information). We propose the remaining MET current is due to TMC2 not downregulating at the same rate in *Tmc1^−/−^* as in the *Tmc1^−/+^* mice suggesting MET current is still present at a lower level at P10 mid‐apex.

We also investigated the *Tmc2^−/−^* mice where the onset of MET is delayed until TMC1 expression begins.^[^ ^54^ ^]^ For *Tmc2^−/−^* mice, hair bundle membrane viscosity, measured at the base between P1‐P4, was high at P1 (880 ± 260 cP for OHC and 690 ± 180 cP for IHC) compared to littermate controls (445 ± 65 cP for OHC and 490 ± 60 cP for IHC) for both IHCs and OHCs (Figure 4E,F and *t*‐test, *p < 0.001*). By P4, viscosity measurements were comparable between the *Tmc2^−/−^* and *Tmc2^−/+^*, consistent with TMC1 expression compensating for the loss of TMC2. These data show a direct link between TMC expression and effective viscosity and are consistent with both TMC1 and TMC2 reducing stereocilia membrane viscosity. Whether these effects are directly due to TMCs acting as scramblases or indirectly impacting scramblase activity through calcium regulation, current or voltage remains to be determined.

### 2.6 MET Channel Blocking Increases Stereocilia Membrane Viscosity

**Figure 5:**
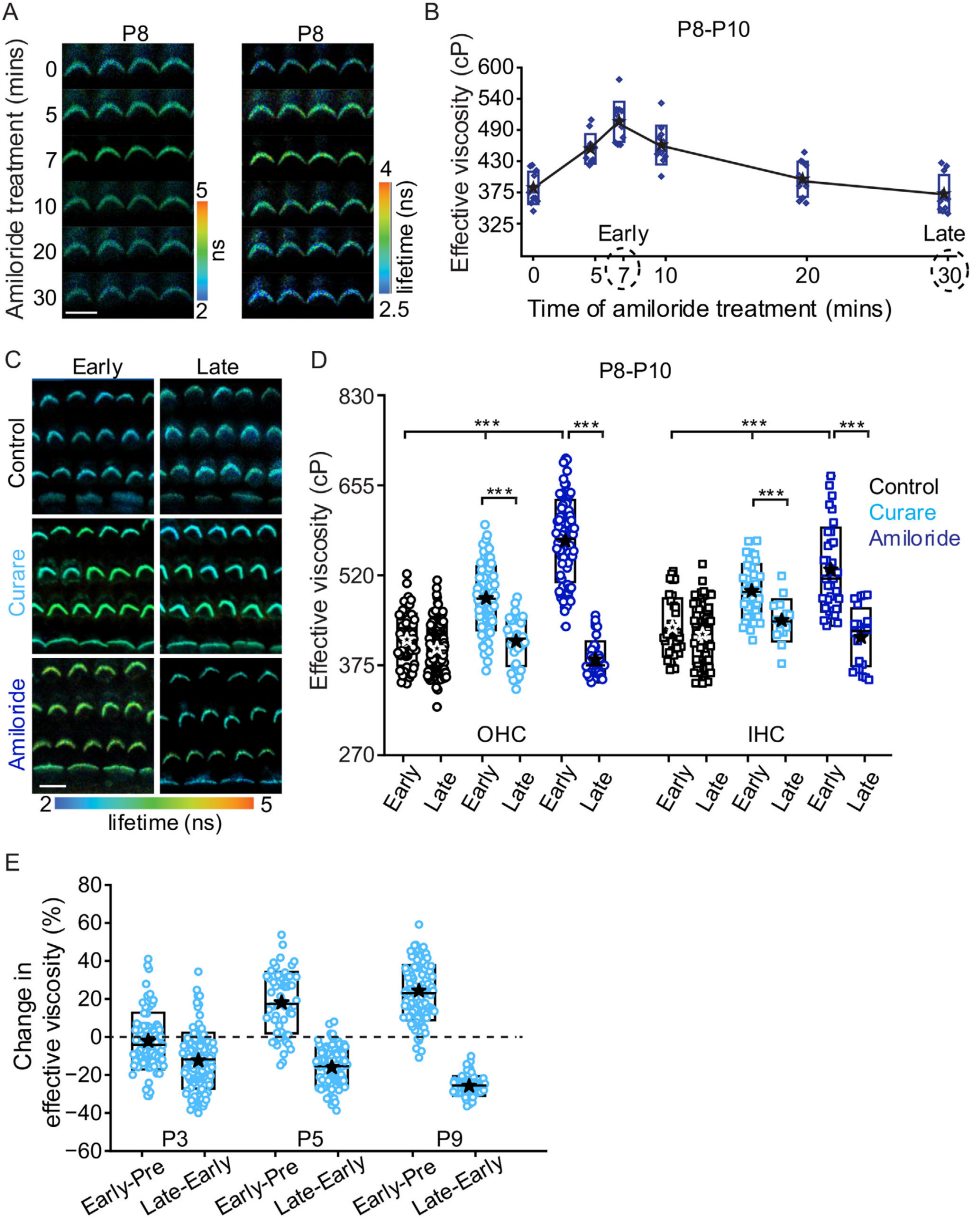
MET channel blocking increases stereocilia membrane viscosity. A) FLIM images of OHCs during a 30‐minute time course where amiloride was continuously applied. B) Quantification of the effective viscosity across samples from hair bundles during amiloride treatment (n = 3 animals). C) Early (≈10 min) and late (≈30 min) FLIM images of hair bundles for each compound. D) Summary of membrane viscosity of hair bundles across preparations and cells for early and late time points from control, curare and amiloride treatments (n = 4 animals for each group). E) Quantification of the percentage change in effective viscosity between early curare and pre‐curare condition and late and early curare treatment in OHCS at P3 (n = 3), P5 (n = 4) and P9 (n = 4) rat apical turns. Boxes in B, D and E represent the SD, and the star symbol indicates the mean and the line indicates the median. ***p < 0.001. Scale bar = 10 µm.

Are the changes in membrane effective viscosity dynamic or established during development? It is possible that membrane properties co‐vary with expression of TMCs during development and that the genetic manipulations interfere with the natural maturation process. We used two known MET channel blockers, tubocurarine (1 mM) and amiloride (1 mM) in P8‐P10 rat pups where hair bundles are mechanically sensitive. Curare is an open channel blocker, while amiloride is a permeable channel blocker thought to bind somewhere within the channel pore.^[^ ^44^, ^45^, ^46^, ^47^, ^48^ ^]^ **Figure** **5**A shows FLIM images of OHC bundles over a 30‐minute time course where amiloride was continuously applied. Hair bundle viscosity initially went up from ≈395 cP, reached the peak near 7 minutes (506 ± 30 cP) and then reduced to near baseline by 30 min (Figure 5B). Figure 5C shows early (≈10 min) and late (≈30 min) FLIM images for each compound with Figure 5D providing a summary of effective viscosity across preparations and cells. Both compounds evoked an initial increase in viscosity (485–497 cP for curare and 530–570 cP for amiloride compared to 415–435 cP for control) followed by a slower recovery to baseline levels in the face of continued drug application (*t*‐test, *p < 0.001*). The initial increase in viscosity suggests the presence of flippases and floppases that are actively creating membrane order which increases viscosity. It further suggests that open MET channels are important for maintaining lower hair bundle membrane viscosities. The increase in effective viscosity indicates a continuous mechanism serving to elevate viscosity that is countered by the MET channel being open. When does this dynamic equilibrium begin? The earliest ages we explored show high effective viscosities, so either membrane order, regulated by flippases/floppases has begun or membrane composition is different at early ages.

We treated both P3 and P5 with curare, choosing an apical region where MET currents had not manifested at P3 but were present at P5. We found an increase in effective viscosity (*t*‐test, *p<0.001*) for OHCs at P5 but not at P3 suggesting the membrane is also being dynamically regulated at the onset of MET (Figure 5E and Figure S6, Supporting Information). Thus, it appears that the mechanism underlying the increased viscosity is established prior to the onset of MET and is sustained after the establishment of MET. It also suggests that both TMC1 and TMC2 are involved in the scramblase activity associated with the MET channels being active.

Another possibility is that the changes measured over time reflect a redistribution of the sensor and not active changes in effective viscosity. One argument against this possibility is that the sensor signal remains stable over the time course of our experiments if we do not intentionally change the environment. Figure 5 shows early and late lifetime measurements are not different from each other in P8‐P9 control for both IHC and OHCs. However, Figure S7, Supporting Information shows there is a decrease in stereocilia intensity and an increase in soma intensity over this same time period. These data suggest that the sensor is trafficked from the stereocilia into the soma. Fortunately, FLIM is independent of concentration and showed no change in effective viscosity.

We further tested whether sensor redistribution impacted effective viscosity changes by using BSA to extract sensor from the external leaflet.^[^ ^2^, ^3^ ^]^ We performed these experiments in two ways, initially we preloaded the sensor so it could enter the hair cells via MET channels and then treated with curare. The second approach was to co‐treat with sensor and curare to prevent sensor from entering the cells via MET channels. Figure S4D,E, Supporting Information presents examples from each condition and provides a summary of the changes in sensor distribution. As expected and similar to the developmental experiments, we found that the majority of sensor was in the inner leaflet, ≈80% in the absence of curare and that ≈55% was in the inner leaflet in the presence of curare. This result supports the conclusion that sensor enters the hair cell via MET channels so blocking MET channels reduces the sensor level at the inner leaflet. Lifetime reduced slightly and is reflected in the effective viscosity values in Figure S4E, Supporting Information. The slight reduction in effective viscosity under control conditions likely reflects leaflet asymmetry.

Preloading the sensor and then treating with curare, followed by extraction at early and late timepoints shows that there is no difference in the extracted percentages indicating that the initial increase and then decrease in lifetime is not a function of sensor redistribution. Preloading the sensor biases the measurement slightly toward the inner leaflet while pretreating with curare biases the measurement slightly toward the outer leaflet. At the timepoint where effective viscosity is increased in the presence of curare, sensor extraction results in a greater reduction in effective viscosity than control. Furthermore the effect is greatest when the sensor reflects the outer leaflet more and so potentially blocking MET channels is increasing the effective viscosity of the outer leaflet. Increased asymmetry is predicted if a scramblase is inhibited while flip/floppase activity continues. In spite of the increased asymmetry, the post extraction effective viscosity in the presence of curare does not reach the level of the control data at the early time point showing that redistribution cannot account for the measured responses. In contrast, the later time point, where effective viscosity has reduced to at or slightly below control baseline, BSA extraction has no effect on the effective viscosity. These data indicate that a compensatory mechanism is evoked that reduces asymmetry between leaflets, likely targeting the outer leaflet as that leaflet appears to have increased its effective viscosity.

There are three key takeaways from the channel blocking experiments. MET channels are involved in regulating membrane viscosity, though whether this is a direct or an indirect action remains to be tested. Second, the increase in effective viscosity at early and late time points supports the presence of an active process, likely flippases and floppases increasing membrane viscosity, counter to the action of the MET channels. Third, the secondary reduction in effective viscosity suggests the presence of a third process working in parallel with MET channels to reduce membrane viscosity, by reducing leaflet asymetry.

### 2.7 PS Externalization Doesn't Drive Membrane Viscosity Change

**Figure 6:**
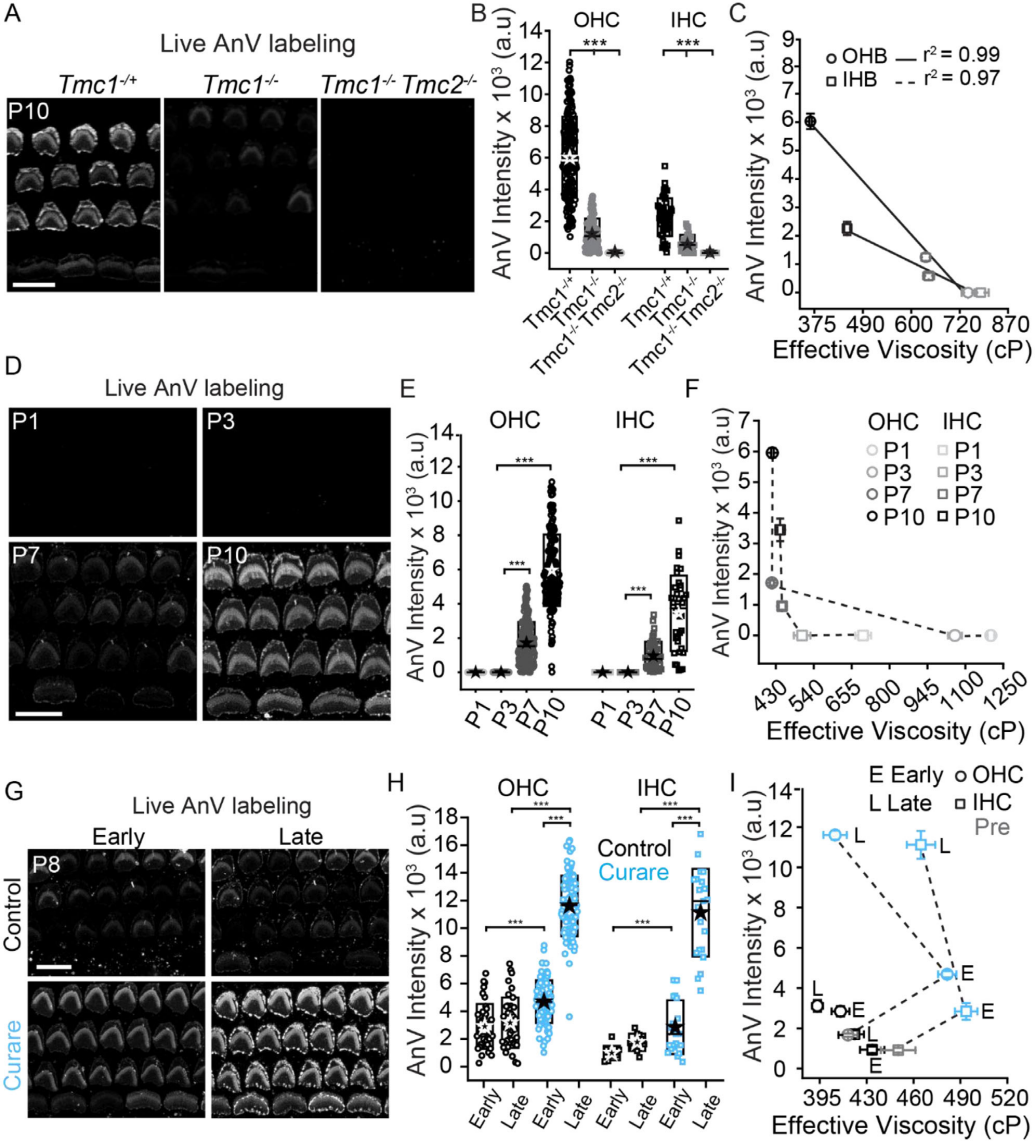
A) Live confocal images of OHCs and IHCs labelled with AnV from mid‐apical turns of P10 *Tmc1^−/+^*, *Tmc1^−/−^* and *Tmc1^−/−^; Tmc2^−/−^* B) Quantification of AnV intensity for OHC and IHC bundles for *Tmc1^−/+^*, *Tmc1^−/−^* and *Tmc1^−/−^; Tmc2^−/−^* (n = 4 animals in each group). C) Plot of mean AnV intensity versus mean effective viscosity measured for OHB (circles) and IHB (squares) of P10 *Tmc1^−/+^*, *Tmc1^−/−^* and *Tmc1^−/−^; Tmc2^−/−^*. D) Live confocal images of OHCs and IHCs labelled with AnV from mid‐apical turns of P1, P3, P7 and P10 rats (n = 5 animals in each group). E) Quantification of AnV intensity for OHC and IHC bundles at different ages. F) Graph obtained by plotting hair bundle AnV intensity on the y‐axis and the hair bundle viscosity measured on the x‐axis for OHC (circles) and IHC (squares) at different ages with darker shade indicating older ages. G) Live confocal images of OHCs and IHCs labelled with AnV from P8 rat mid‐apical turns untreated (control) or treated with 1 mM curare for ≈10 min (early) or ≈30 min (late) H) Quantification of AnV intensity for OHC and IHC bundles treated as in G (n = 3 animals for control group and n = 4 animals for curare treated group). I) Graph obtained by plotting AnV intensity on the y‐axis and the viscosity measured on the x‐axis showing no correlation between both. Boxes in B, E and H represent the SD, and the star symbol indicates the mean and the line indicates the median. Symbols in C, F, and I represent the mean and the error bars indicate the SE. ***p < 0.001. Scale bar = 10 µm.

TMC molecules are part of a superfamily of proteins including the TMEM16 and OSCA/TMEM63 molecules which can be ion channels and/or lipid scramblases.^[^ ^34^ ^]^ As externalization of PS, which is usually restricted to the inner leaflet, is often used as a marker for scramblase activity,^[^ ^7^ ^]^ we used the PS‐specific binding protein Annexin V (AnV) labeling to detect PS externalization. Emerging evidence suggest that PS externalization via TMEM16F lipid scramblase can reduce membrane viscosity.^[^ ^3^, ^9^ ^]^ In hair cells, recent evidence suggests that PS externalization triggered by MET channel inhibition requires TMCs.^[^ ^35^, ^36^ ^]^ We first addressed whether the high membrane viscosity in *Tmc1^−/−^* and *Tmc1^−/−^ Tmc2^−/−^* mice correlates with a reduction in PS externalization (due to a lack of scramblase activity). At P10, following a 10 min incubation with AnV, the littermate controls showed a range of live AnV labeling that was significantly greater (*t*‐test, *p < 0.001*) than in the *Tmc1^−/−^* which was also significantly greater (*t*‐test, *p < 0.001*) than in the *Tmc1^−/−^ Tmc2^−/−^* double KO (raw images shown in **Figure** **6**A and summarized in Figure 6B). We are suggesting this labeling is not an indicator of cell's being compromised, but rather as yet another mosaic region associated with cell maturation. One reason for this is that the AnV labeling does not increase during the time of recording for control experiments (Figure 6G,H) Higher hair bundle viscosity (from Figures 2 and 4) and lower AnV labeling in these mutants correlated with a reduction in the presence of TMC molecules (Figure 6C). Both IHC and OHC showed strong negative correlations between parameters but were different from each other (OHB: r^2^ = 0.99, *p* < 0.001, IHB: r^2^ = 0.97, *p* < 0.001). These data support the hypothesis that the high membrane viscosity in the TMC mutants could be due to reduced scramblase activity from the lack of TMC activity. It is also possible that the lipid composition in these mutants is different from their WT controls and that this contributes to higher membrane viscosity.

We then tested whether changes in hair bundle effective viscosity during development are due to the lipid scramblase activity associated with the MET machinery. Live AnV labeling (post 10 min incubation) from the mid‐apical region of P1, P3, P7 and P10 rats showed AnV labeling for both IHC and OHC bundles increasing from P7 (Figure 6D,E). However, when correlating AnV labeling with the change in membrane viscosity in Figure 3, most of the viscosity changes occur prior to the increase in AnV labeling, that is, before P7. Additionally, the AnV labelling continues to increase after the membrane viscosity has stabilized between P7 and P10 (Figure 6F), yielding no correlation between membrane viscosity and the classic scrambling signal, that is, PS externalization. This suggests that PS externalization is not the major underlying cause for the reduction in membrane viscosity observed during development. As PS is not the only lipid transported, it is possible that other lipids such as PE are scrambled earlier than PS. Currently there are no reliable markers for other lipids.^[^ ^62^, ^63^, ^64^ ^]^

The AnV labelling at P7/P8 (Figure 6D,G) and P5 (Figure S6C, Supporting Information) appears to target the kinocilium more than the rest of the hair bundle. At P8, the kinocilium is in the process of being removed from the hair bundle. The preferential labelling of kinocilia with AnV suggests PS externalization serves multiple roles and simply using AnV and interpreting its distribution to suggest cells are compromised is at best an oversimplification of the biology. Kinocilia AnV labelling was validated by co‐labelling AnV with microtubule antibody (Figure S8, Supporting Information). These data are from P9 so there are fewer hair bundles with kinocilia, however those with kinocilia show colocalization. Most of the hair bundles no longer have kinocilia. Other cells, like pillar cells show microtubule labeling consistent with a primary cilium and there is no AnV labeling on those protrusions, likely because they are not in the process of being removed.

As curare treatment initially increased effective viscosity (Figure 5), we tested whether this increased viscosity was due to a reduction in PS externalization. Surprisingly, curare treatment resulted in an increase in the AnV labelling at both the early time‐point where effective viscosity was elevated and at the later time‐point where effective viscosity was reduced for both OHC and IHC bundles at P8‐P10 (Figure 6G–I and *t*‐test, *p < 0.001*). The increase in the AnV labelling at later time‐point is consistent with previous studies^[^ ^35^ ^]^ but unexpected at the early time point as effective viscosity is increasing and MET channels are blocked. Unlike effective viscosity, AnV labeling increased at both the early and late time points (Figure 6I). These data suggest that channel block triggered PS externalization either directly or indirectly. It also suggests that either TMCs can scramble even when the MET channel is blocked, or a second scramblase is activated when MET channels are blocked. At P3, where MET currents are not active and effective viscosity is high, there was no PS externalization either in control or in the presence of curare, even at late time points (Figure S6C, Supporting Information).

The PS data is consistent with the conclusions drawn from curare experiments suggesting that the membrane composition is directly changing over the time course where effective viscosity first elevates and then decreases. The initial increase in outer leaflet viscosity appears to be happening in spite of PS externalization increasing and potentially the greatly increased PS externalization is to equilibrate the membrane in the face of MET channel closure. The data indirectly support the presence of an active flippase/floppase and directly support the presence of a scramblase. For the MET channel to be the scramblase activated by blocking MET current flow would require the selection of lipids to be scrambled to shift to PS because scrambling was happening prior to MET channel block and PS was not the major lipid being scrambled. Alternatively, a second scramblase is activated when MET activity is reduced.

### 2.8 Increase in Stereocilia Membrane Viscosity is Independent of MET Current and Ca^2+^ Entry

**Figure 7:**
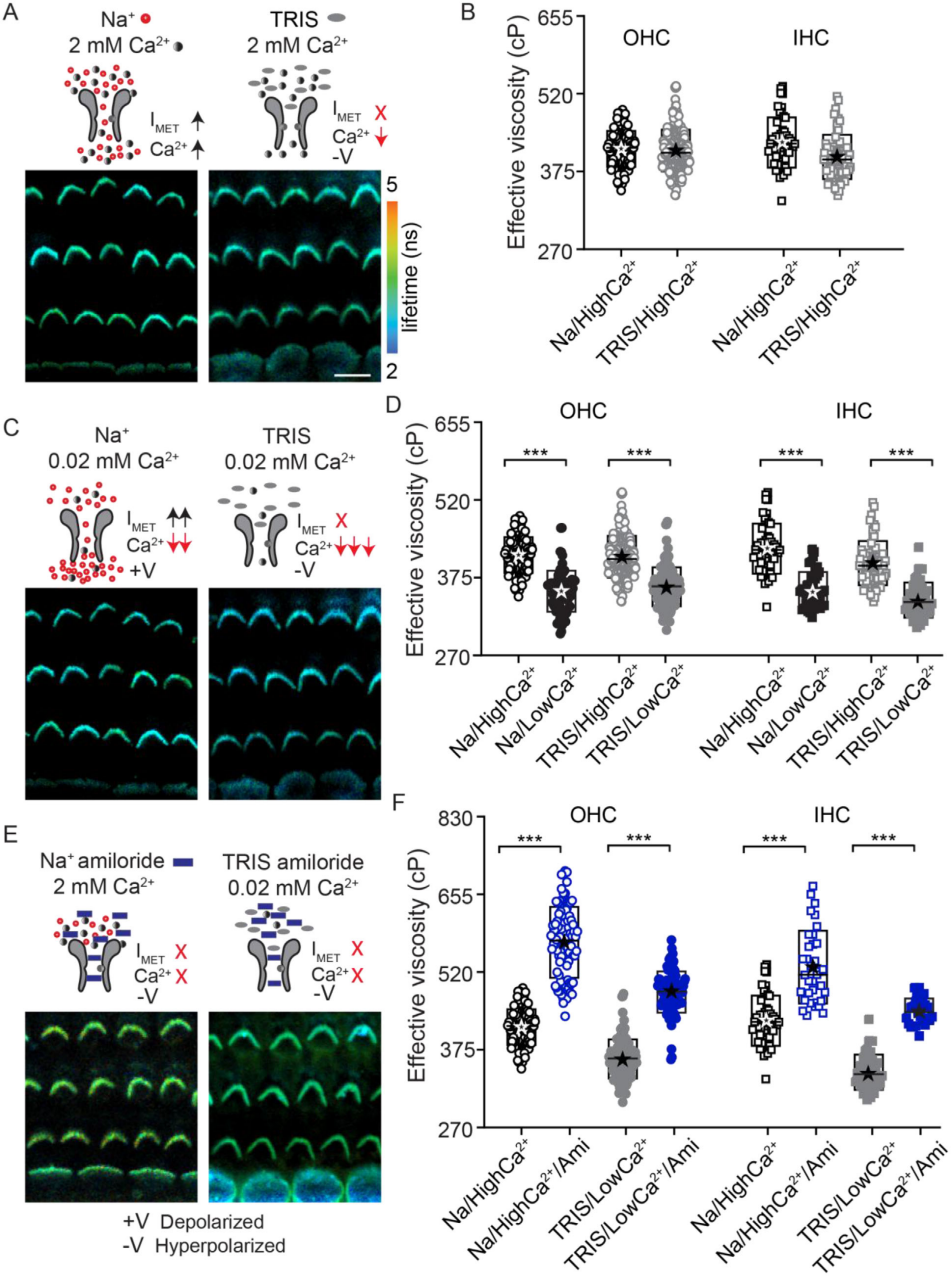
Increase in stereocilia membrane viscosity is independent of MET current and Ca^2+^ entry. FLIM images from P10 rat mid‐apical organ of Corti with A) different monovalent ions (Na^+^ versus TRIS^+^) and 2 mM Ca^2+^, C) different monovalent ions (Na^+^ versus TRIS^+^) and 0.02 mM Ca^2+^ E) different monovalent ions (Na^+^ versus TRIS^+^), different Ca^2+^ concentrations (2 mM versus 0.02 mM) in the presence of 1 mM amiloride. The manipulations are represented schematically above each representative FLIM image. B,D,F) Summary plot showing the effective viscosity measured for the hair bundles from each manipulation stated above. Each manipulation was performed in at least 4 animals. Boxes in B, D, and F represent the SD, and the star symbol indicates the mean and the line indicates the median. ***p < 0.001. Scale bar = 10 µm.

At this point, PS labeling coupled with viscosity measurements show the membrane modulation is complex, likely reflecting multiple processes. We and others have not yet demonstrated that TMC's were scrambling as opposed to regulating other scramblase activity indirectly through membrane potential or calcium. To address whether MET channels also scramble, we biophysically separated the MET channel state from its effectors, namely calcium and membrane potential. Current through the MET channel regulates the membrane potential, such that blocking the channel hyperpolarizes the cell potentially triggering a voltage dependent effect (deactivating scramblase activity or activating flippases and floppases). Ca^2+^ through the MET channel could be driving some secondary effect (like above) or the blocker when in the pore may block both MET current and TMC scramblase activity that in turn unmasks independent flippase/floppase activity to increase membrane viscosity. To directly address these potential processes, we took advantage of known MET channel pore properties to manipulate channel permeation.^[^ ^45^, ^65^, ^66^ ^]^ **Figure** **7**A,C,E summarizes the manipulations used schematically and presents corresponding hair bundle lifetime images. In our control extracellular solution with Na^+^ as the major monovalent ion and [Ca^2+^] at 2 mM, MET currents are large, despite Ca^2+^ blocking more than 50% of the current and carrying about 60% of the current.^[^ ^66^ ^]^ When Na^+^ is replaced with an impermeant monovalent ion TRIS^+^, the total current is reduced by more than 50% and Ca^2+^ current also reduces because without a permeant monovalent ion, Ca^2+^ inhibits its own permeation.^[^ ^66^ ^]^ Thus, there is a large total reduction in MET current and a modest reduction in Ca^2+^ entry. Hair cells are expected to hyperpolarize. This manipulation resulted in no statistical difference (*t*‐test, *p>0.05*) in membrane viscosity suggesting that neither current nor voltage were responsible for the effect on membrane seen with the channel blocker (Figure 7A,B). Lowering external Ca^2+^ increases the total MET current by reducing the Ca^2+^ block of the channel and increases the resting open probability of the channel through a slow adaptation process.^[^ ^66^, ^67^, ^68^ ^]^ Together these changes are expected to depolarize hair cells.^[^ ^45^ ^]^ Lowering Ca^2+^ to 20 mM while maintaining Na^+^ as the monovalent resulted in a decrease in viscosity (415–430 cP at 2 mM to 350–355 cP at 20 mM; Figure 7C,D; *t*‐test, *p<0.001*). This decrease in viscosity could be due to any of the factors described above. To isolate the MET channel being open from both Ca^2+^ and depolarization, we used a TRIS^+^ and 20 mM Ca^2+^ solution. In this case, MET channels open, however there will be significantly less total current and even less permeation by Ca^2+^, resulting in the hair cells hyperpolarizing despite the channels being open.^[^ ^66^ ^]^ In this case, stereocilia viscosity reduced for both IHCs and OHCs (390–415 cP at 2 mM and 340–360 cP at 20 mM; Figure 7C,D; *t*‐test, *p < 0.001*). Thus, neither Ca^2+^ nor current (or membrane potential) are responsible for the lower viscosity, rather the increased open probability of the channel likely reflects an increased scramblase activity. Adding amiloride to the TRIS^+^ and 20 mM Ca^2+^ solution confirms our result as viscosity increased in the presence of the channel blocker despite there being no change in current or Ca^2+^ entry (340–360 cP w/o amiloride and 445–480 cP with amiloride; Figure 7E,F; *t*‐test, *p < 0.001*). Given that under these circumstances there is little effective current or Ca^2+^ permeation, the increased viscosity is most likely ascribed to the channel blockers directly inhibiting scramblase activity. The relative change in lifetime between control solution and amiloride application (Δ160 for OHC and Δ95 for IHC) and the TRIS^+^ + 20 mM Ca^2+^ solution and TRIS^+^ + 20 mM Ca^2+^ + amiloride (Δ120 for OHC and Δ105 for IHC) are comparable, suggesting that the underlying mechanism is the same and independent of current and Ca^2+^. Together these data represent the first direct evidence that the MET channel state regulates membrane viscosity.

## 3 Conclusion

Our data provides the first evidence that stereocilia membrane viscosity is directly regulated by the MET channel complex in cochlear hair cells. Data further suggest a yet undefined system creating a highly viscous membrane that is then regulated by the MET channel's activity. We suggest the combination of mechanosensitive scramblase activity coupled with local flippase and floppase activity provides an energy‐dependent homeostatic feedback mechanism that modulates force translation to the MET channel complex.

**Figure 8:**
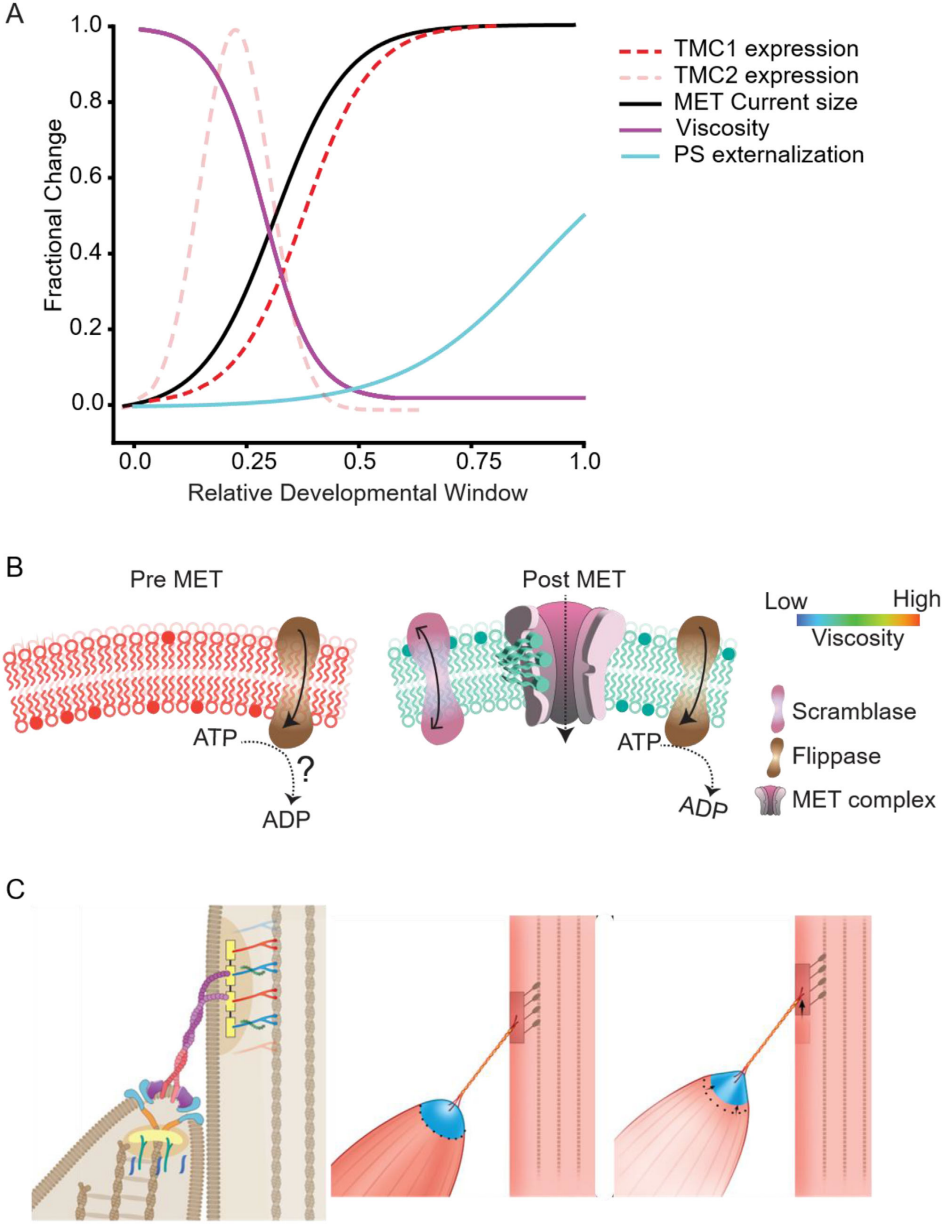
Dynamic modulation of auditory hair cell stereocilia membrane mechanics by the MET complex. A) Schematic summarizing the relative changes of different hair bundle properties during early postnatal developmental age. B) provides a schematic of the membrane regulating molecules identified in the present study. C) Depending on how the various components of the MET machinery are mechanically coupled together, the impact of the plasma membrane on the force translation pathway will be determined by whether the membrane is more in series or in parallel.

Several pieces of evidence target the MET complex as key to the reduction in membrane viscosity. First is the strong correlation between the developmental onset of MET current, and stereocilia membrane viscosity. Second is deafness‐causing MET channel mutants, *Tmc1^−/−^, Tmc2^−/−^*, *Tmc1;Tmc2* double mutant and *Tmie^−/−^*, showing higher viscosity compared to their littermate controls. Third, MET channel blockers increase membrane viscosity under conditions where the channels are open, but current was not flowing; thus, ruling out the effects of current, voltage or Ca^2+^ and providing direct evidence that the opening of the MET channels is reducing the membrane viscosity, potentially through their scramblase activity that could be inhibited by these same channel blockers. The concomitant increase in effective viscosity in the presence of the channel blocker provides evidence for the presence of active flippase/floppase throughout the developmental time window. They are also functionally identified pre‐onset of MET current and their presence remains at least until postnatal day 10 (our last measurement point). Scramblase activity opposes the flippase/floppase scenario to create a dynamic equilibrium where the stereocilia are at a lower effective viscosity than if there were no scramblase but higher than if there were no flippase/floppase. **Figure** **8**A summarizes the relative changes in MET current amplitude, membrane viscosity, the presence of TMC 1 and TMC2 as well as PS externalization.

### 3.1 Active Lipid Transport

This study suggests there is a dynamic interplay between scramblase activity sensitive to the MET open probability and an independent mechanism serving to increase membrane effective viscosity (Figure 8B). We hypothesize flippase/floppase activity is involved from the very high effective membrane viscosity prior to the onset of MET, in the mutant mice that eliminate the presence of the MET complex or the TMC components of the complex and with blockers of the MET channel resulting in increased effective viscosity. The high viscosity could also be attributed to lipid diffusion when the scramblase is blocked, different lipid compositions being present during development and membrane interaction with underlying cytoskeletal structures.^[^ ^69^, ^70^ ^]^ Significant developmental changes in membrane properties have been previously reported in other systems and associated with lipid composition.^[^ ^71^, ^72^, ^73^ ^]^ However, the highest viscosity reported in our study is similar to that reported for model membranes made entirely of sphingomyelin and cholesterol, one of the most viscous lipid combinations known and a composition unlikely to be found in stereocilia at any age. Additionally, the lipid composition cannot account for the increased effective viscosity following MET channel blocker application as early as postnatal day 5. The increased effective viscosity is consistent with an enhanced membrane order typically achieved by active transport via flippase or floppase activity. If effective viscosity were not dynamic, the channel blocking experiments would predict no viscosity change. The stereociliary membrane specifically expresses ATP8B1 flippase that transports PS from the outer to the inner membrane. Morphological and functional degeneration of the hair bundles due to the mutations of ATP8B1 and ATP8A2 indicate the importance of this transport mechanism involved in maintaining the stereocilia.^[^ ^74^, ^75^ ^]^ However, recent ongoing studies indicate that ATP8B1 flippase starts expressing in the stereocilia from post‐natal day 7. This timing is consistent with our AnV labeling but suggests an additional pathway, perhaps ATP8A2 which is present in hair bundles as early as P0.

Previous work using AnV labeling suggest that TMC1 and not TMC2 is required for PS externalization in mammalian hair cells.^[^ ^35^ ^]^ Our work suggests that both TMC1 and TMC2 are directly involved in stereocilia membrane regulation. Effective viscosity reduces during the time when TMC2 is present and the loss of either TMC1 or TMC2 elevates effective viscosity. Modulating MET channel open probability while reducing current flow and calcium entry directly implicate MET channel state with scramblase activity, ruling out membrane potential and calcium as driving the scrambling process. We do not rule out that calcium and voltage are modulating other components of the process on different time scales.

### 3.2 PS Externalization as a Marker for TMC Activity

The correlation of PS externalization with effective viscosity and TMC activity is complex and indirect. Both developmental data showing an early decrease in effective viscosity when TMC2 is being expressed and the *Tmc1* ^−/−^ mouse showing a decrease in effective viscosity are consistent with TMC2 having scramblase activity. Developmental reductions in viscosity do not correlate with PS externalization until P7 and later. Previous work also suggested that Ca^2+^ buffering was activating scramblase activity. Our study shows that blocking the MET channel leads to an initial increased viscosity which cannot be explained by activating a scramblase, however PS externalization increases in this time frame suggesting a scramblase is activated. At later times effective viscosity is reducing and AnV labelling continues to increase. The latter is consistent with the previous reports.^[^ ^35^ ^]^ The viscosity sensor demonstrates though that PS labeling is limited in that it is not clear that all scramblases move PS equally or perhaps that PS is not present early in development. The kinocilia labeled by PS at P5 and P7 support the conclusion that PS externalization has multiple functions. There are several possible explanations for the early mismatch between effective viscosity and PS externalization. First, a second scramblase might be activated when MET channels are blocked and this scramblase is more selective for PS. Second, PS at the inner leaflet might be increased when MET channels are blocked and the scramblase activity of TMCs might not be blocked. This would lead to increased PS externalization. Third, blocking MET channels might alter the selectivity of the MET scramblase such that it shifts toward preferentially scrambling PS. One might envision that blocking the MET channel alters the local membrane mechanics as slow MET adaptation process try to open more MET channels which would then be blocked shifting the adaptation response further and increasing local membrane tension. This change in tension might change scrambling properties to select for PS. Each of these are worthy of further investigation.

### 3.3 Scramblase Speed

Are the molecules associated with the MET channel capable of altering the effective membrane viscosity of the entire stereocilia, including row 1 stereocilia where MET channels have not been identified? Recent evidence of TMEM16F scramblase rates suggests up to 45 000 lipids per second.^[^ ^76^ ^]^ A stereocilium modelled as a cylinder of 0.4 µm diameter and a height of 4 µm^[^ ^77^ ^]^ produces a surface area of ≈5.3 µm^2^. Previous reports indicate an average surface area for each lipid molecule of ≈0.5 nm^2^,^[^ ^78^, ^79^, ^80^ ^]^ and assuming two functional channels in a stereocilium,^[^ ^15^, ^81^ ^]^ we can estimate it would take 3.9 min for the channel to turnover its entire stereocilium membrane. It has been suggested that there are more TMC molecules per stereocilia than account for 2 MET channels and if these contribute to scrambling, the time to turn over the stereocilia would be much shorter.^[^ ^60^ ^]^ Finally, this rough calculation does not include membrane proteins that would reduce the lipid concentration and further speed up membrane turnover.

### 3.4 Functional Relevance

The present work identifies a dynamic regulation of stereocilia membrane mechanical properties that is regulated by mechanically‐sensitive MET channel complex and a mechanism increasing membrane effective viscosity. Given that hair cell MET channels operate at the highest known rates of any mechanoreceptor system and has both measured and calculated sensitivities at molecular dimensions. A simple idea is that reducing membrane viscosity will lower the energy for channel gating which may enhance both the frequency and sensitivity of the response.^[^ ^82^, ^83^, ^84^ ^]^ If speed was the sole purpose though, active flippase/floppase activity would not be required. Our data suggest that the membrane is being actively regulated and this process is sensitive to the MET channel open state and so is also mechanically sensitive. When the MET channel opens effective membrane viscosity will reduce. If reducing membrane viscosity leads to channel closure, one can envision how this might contribute to say the fast adaptation response, where local viscosity changes could happen in the milli to sub millisecond time frame. As fast adaptation has been measured in the 10s to 100s of microseconds, is it feasible that effective viscosity can work that fast? Central to answering this question is determining how much plasma membrane is involved in the force translation process. Previous computational data suggests a disc of diameter 100 nm is sufficient to distribute force from the tip‐link to MET channels with enough energy to gate channels but not enough to pluck the channel from the membrane. This disc has also been shown to have mechanical properties that might be consistent with gating compliance and responses elicited by flexible fiber stimulation. Recent high‐resolution imaging of myosin XV presents rings at the stereocilia tips of ≈80 nm providing some indirect evidence for this disc construct. Using the previous scramblase rates even without accounting for surface areas associated with transmembrane proteins predicts complete membrane turnover in less than 100 ms assuming 2 scramblases. So it is plausible that fast adaptation is a result of changes in force at the MET channel resulting from changes in effective viscosity. If on the other hand lowering viscosity opens channels, then one might envision co‐operativity between channels like what has been predicted computationally.^[^ ^26^ ^]^ More direct measurements, outside the scope of present work, are needed to sort this out.

Another way to envision the membrane impact on the MET process is to consider the complexity of the molecular coupling of the machinery (Figure 8C). Previous studies have reported on the existence of multiple molecular interactions between the MET channel components and the lower part of the tip link, PCDH15. For example, TMC1, TMC2, TMIE, LHFPL5, LOXHD1 and Myo15 can interact with PCDH15 and each of these molecules also interacts directly with the membrane.^[^ ^85^, ^86^, ^87^, ^88^ ^]^ Depending on how this machinery is wired together, the plasma membrane might be considered in series and/or in parallel with the force translation pathway. Figure 8C depicts that the membrane tenting when the tip link is pulled will interact with the various components of the MET machinery differently depending on the mechanical coupling and that this could be direct or indirect. The relative contribution of the plasma membrane will depend in part on the mechanical coupling between these molecules, but the impact of membrane viscosity changes will be determined by whether the membrane is more in series or parallel with these elements. If in parallel, a reduction in effective membrane viscosity would be expected to shift more of the force stimulation onto the channel, allowing it to open faster and in response to a smaller total force stimulus. Increasing effective viscosity would result in the channels receiving less of the total force, requiring larger stimulations for channel gating and resulting in slower responses. If in series, force would be the same, however motion would change with the lower effective viscosity resulting in larger motion (i.e., spring extension).

And finally, as the scramblase is using energy likely provided by the flippase activity that establishes an electro‐lipid gradient across the bilayer, it is plausible to imagine this system contributing to hair bundle amplification.^[^ ^83^ ^]^ Given the known rates of flippase/floppase and scramblase activity, this active mechanically regulated feedback system better fulfills the needed requirements for cycle‐by‐cycle hair bundle amplification than do constructs invoking myosins or needing calcium diffusion. Potentially, a combination of mechanisms are used to create a standing tension translated to the local MET channels and the MET channels use and regulate the stored energy by regulating lipid order. Thus, this newly identified membrane regulation that is potentially established by the interplay of flippases/floppases and scramblases that are also mechanically sensitive ion channels provides a new framework from which to evaluate old and new data about hair bundle mechanics and MET channel properties.

## 4 Experimental Section

### Sample Preparation

*Cochlear Explants*: All animal experiments were approved by the Stanford University Administration Panel on Laboratory Animal Care (APLAC #14 345). Rodents (rats and mice) of both sexes were used in the experiments. The wild‐type (WT) C57BL6/J mice and Sprague‐Dawley rats were purchased from Charles River Laboratories. *Tmie^KO^* ^[^ ^52^ ^]^ (B6.B(CBA)‐*Tmie^sr^*/J, JAX 000543) was obtained from JAX, *Tmc1^KO^* ^[^ ^54^ ^]^ (B6.129‐*Tmc1^1.1Ajg^*/J, JAX 01 9146, backcrossed on C57Bl6/J) from Dr. Beurg and Dr. Fettiplace (Wisconsin University) and *Tmc2^KO^* from Dr. Angela Ballesteros.^[^ ^35^ ^]^ For mice, heterozygous littermates served as controls. Experiments were performed blinded to genotype. The mice were genotyped as previously described.

Animals were sacrificed by decapitation and cochlear turn of isolated organ of Corti was dissected from pups at postnatal day 1 (P1) to P10 and placed in a recording chamber as described previously.^[^ ^15^ ^]^ Briefly, temporal bones were removed from the skull and cochlear tissue was placed in extracellular solution containing (in mM): 142 NaCl, 2 KCl, 2 CaCl_2_, 1 MgCl_2_, 10 HEPES, 6 Glucose, 2 Ascorbate /Pyruvate, 2 Creatine monohydrate, at pH 7.4 and a final osmolality of 304 – 307 mOsm. After removing the semicircular canal and vestibular organs, the cochlear bone was removed to expose the organ of Corti. The modiolus, Reissner's membrane, and the tectorial membrane were sequentially removed. The tissue was then incubated with 10 µM of BODIPY (boron dipyrrin) 1c in extracellular solution at room temperature (RT) for 6 min, protected from light. BODIPY 1c stock solution (100 mM) was prepared in 100% ethanol and stored at −20 ^°^C. Working dye solution was made daily by diluting in extracellular solution to a final BODIPY 1c concentration of 10 µM in 0.01% ethanol and kept at room temperature (RT; 19–21 °C), protected from light. In a few experiments, the tissue was incubated in PS‐binding AnV‐Alexa Fluor 647 (1:50 dilution in extracellular solution) for 10 min at RT.

The tissue was then transferred to the recording chamber with dye‐free extracellular solution and held in place with single strands of dental floss while ensuring that inner hair cell (IHC) bundles were oriented vertically. During the experiment, tissue was perfused with extracellular solution at rate of 0.3 mL min^−1^ maintained at RT. In back‐extraction experiments, the tissue was incubated in 1% fatty acid free Bovine Serum Albumin (BSA, A4612, Sigma)) in external solution for 2–3 min.

### Fluorescence Lifetime Imaging

FLIM experiments were carried out using an upright Leica TCS‐SP8 FALCON (FAst Lifetime CONtrast) confocal microscope using LAS X version 3.5.7 and LAS X FLIM/FCS 3.5.6 softwares (Leica Microsystems GmbH, Germany) and an HC APO 20× / 1.0 NA water dipping objective (Leica Microsystems GmbH, Germany). The excitation source was a pulsed white light laser (WLL) with an acoustic‐optical beam splitter (AOBS). BODIPY 1c was excited at 480 nm and the pulse repetition frequency of the laser was set to 40 MHz. Two sensitive hybrid detectors (SMD, Leica Microsystems CMS GmbH, Germany) allowed for photon detection from 490–560 and 600–670 nm. A frame accumulation of 50 was set to allow better photon statistics. The laser intensity was set to keep the photon count of ≥ 1 per laser pulse to avoid photon “pile up” that leads to shortening of the fluorescence lifetime (Suhling et al., 2012). Z‐stacks were captured at 1 um step interval.

### FLIM Analysis

All FLIM images were analyzed using LAS X FLIM/FCS version 3.5.6 software. Each ROI consisted of a hair bundle on a single z‐plane. Pixels were binned to have at least 300 photon counts at the peak of the decay for reliable analysis. The fluorescence decay curves were fitted using n‐exponential reconvolution with IRF (instrument response function) model. The function of this model is

(1) $$\begin{array}{ccccc} y\left(t\right) & = & \left\{IRF\left(t+Shift_{IRF}\right)\right\} & ⊗ & \left\{\sum_{i=0}^{n-1}\;\;A\left[i\right]e^{\left(-\frac{t}{\tau \left[i\right]}\right)}+Bkgr\right\} \end{array}$$

where A is the amplitudes, τ is the lifetimes, n is the number of exponential components, *Bkgr* is the tail offset (correction for background), and *Shift_IRF_* is the IRF shift (correction for IRF displacement). For the hair bundles, the decay curves were best fitted by biexponential model with the goodness of fit parameter (χ^2^) >1. The average of the two intensity‐weighted lifetime components were calculated by

(2) $$\tau_{Av,Int}=\frac{\sum_{k=0}^{n-1}I\left[k\right]\tau \left[k\right]}{I_{Sum}}$$

where I_sum_ is the sum of the fluorescence intensity of all components.

### Drug Treatment

*MET channel blockers*: In a subset of experiments, during and post BODIPY 1c and AnV staining, the tissue was incubated in 1 mM tubocurarine (93 750, Sigma) or 1 mM amiloride (0890, TOCRIS) to block MET channel.

### Electrophysiology

In several experiments, FLIM was combined with whole‐cell patch clamping to measure MET and membrane viscosity from the same hair bundle. The tissue was viewed in brightfield using a custom built fixed‐stage upright BX 51WI (Olympus) microscope with a digital Rolera‐XR camera (QImaging). Orientation of pipettes and access to the hair cells were as previously described.^[^ ^89^ ^]^ Whole‐cell patch clamp recordings were obtained using thick‐walled borosilicate patch pipettes (WPI) pulled on a P97 micropipette puller (Sutter Instruments) to 1–2 µm inner diameters (3–3.5 MΩ). The internal solution contained (in mM): 116 CsCl, 3.5 MgCl_2_, 3.5 ATP, 5 creatine phosphate, 1 Tetracesium BAPTA, 10 HEPES, and 20 ascorbic acid, pH of 7.2 and osmolality of 285 – 290 mOsm. Extracellular solution was delivered through a bath solution and also through an apical pipette of tip size ≈200 µm.^[^ ^21^ ^]^ The apical perfusion pipette prevented internal solution from reaching BAPTA sensitive hair bundles.

Outer hair cells were voltage‐clamped cells at −80 mV and adjusted offline for liquid junction potential (4 mV). Mechanically elicited currents were low pass filtered at 10 kHz, with an Axopatch 200b patch clamp amplifier (Axon Instruments), sampled at 100 kHz using Personal DAQ3000 (IOtech), and recorded with jClamp (SciSoft). Uncompensated series resistance (Rs) was between 6 and 10 MΩ.

### Mechanical Stimulation

Hair bundles were deflected with a custom‐built fluid jet system as previously described.^[^ ^61^ ^]^ Thin‐walled borosilicate pipettes (10 µm tip diameter) were filled with extracellular solution and positioned within 5 µm of the voltage‐clamped hair bundle. The fluid jet was driven with a 50 Hz sinusoidal wave using a piezo electric disc bender whose input was filtered with an 8‐pole Bessel filter (Frequency Devices) at 1 kHz before and then amplified by a high voltage/high current custom‐built amplifier.

### Immunofluorescence Staining and Imaging

In a few experiments, following the FLIM experiment, some cochlear turns were stained for kinocilia. The dissected sample was fixed in 4% PFA in extracellular solution for 40 min at RT. The fixed samples were washed with PBS 3 times and transferred to a glass well plate with PBS containing 0.05% Triton X‐100, permeabilized for 40 min at RT. After permeabilization, the samples were blocked in PBS with 0.05% Tween 20 (PBST) containing 4% bovine‐serum albumin Fraction V (BSA) overnight at 4 °C. The tissues were then incubated with primary antibody (CST, Acetyl‐α‐Tubulin (Lys40) (D20G3) XP Rabbit mAb #5335, 1:500) in PBST with 1% BSA (incubation buffer) overnight at 4 °C. The samples were then washed four times, 5–10 minutes per wash, in incubation buffer at RT. Then the tissues were incubated with fluorescent dye conjugated secondary antibodies (Donkey anti‐Rabbit IgG with Alexa Fluor 488, Invitrogen A21206, 1:500) in incubation buffer at RT for 1–2 h. Then the samples were washed three times, 5–10 minutes per wash, with incubation buffer. The glass well plate was on a horizontal shaker with a 60‐rpm speed during permeabilization, incubation and washing steps.

After washing, the tissue was transferred to the recording chamber with PBS and held in place with single strands of dental floss while ensuring that the hair bundles were oriented vertically. Z‐stacks were captured using the Lightning super‐resolution mode of an upright Leica TCS‐SP8 confocal microscope with an HC PL APO 63× / 1.20 water immersion objective lens and LAS X 3.5.7 software. Acetyl‐α‐Tubulin were excited using a white light laser that can be tuned for wavelengths between 470 nm – 670 nm. The image acquisition parameters were determined as the best X*Y and Z axis resolution possible for the shortest wavelength used.

### Data Analysis

We used ImageJ for the intensity measurements of BODIPY 1c in the hair cell soma, and the hair bundle intensities measurements of AnV and tubulin. Whole cell currents were visualized and analyzed using jClamp (SciSoft) and OriginPro 2018 (OriginLabs). Graphs were generated with OriginPro 2018 (OriginLabs) and Adobe Illustrator CS6 (Adobe). All statistical analyses used two‐sample Student's *t*‐test performed using OriginPro 2018 (OriginLabs). Significance (*p* values) are * *p* < 0.05, ** *p* < 0.01, *** *p* < 0.001. Data are presented as mean ± SD.

## Conflict of Interest

The authors declare no conflict of interest.

## Author Contributions

S.S.G. and A.J.R. designed the experiments, performed the experiments, analyzed the data and wrote the manuscript.

## Supporting information



Supporting Information

## Data Availability

The data that support the findings of this study are available from the corresponding author upon reasonable request.
