# Supplementary material for: Auditory Hair Cell Mechanotransduction Channels Dynamically Shape the Mechanical Properties of Their Membrane Environment
Source: Adv Sci (Weinh). 2025 Sep 4;13(7):e08268. doi: 10.1002/advs.202508268 (PMC12866819; doi:10.1002/advs.202508268)
Supplement: Supplementary file 1 — Supporting Information [file ADVS-13-e08268-s001.docx]

Supporting Information

Auditory hair cell mechanotransduction channels dynamically shape the mechanical properties of their membrane environment

Shefin George*, Anthony. Ricci*

**Validation and calibration of BODIPY 1c**

Molecular rotors are small synthetic fluorophores that exhibit viscosity-dependent fluorescence quantum yield and fluorescence lifetime i.e., the average time a fluorophore remains in the excited state (Dent et al., 2015; Kuimova et al., 2008; López-Duarte et al., 2014; Sherin et al., 2017). Rotation or intramolecular twisting of the molecular rotor leads to non-radiative decay from the excited state back to the ground state. In an ordered or more viscous membrane, the non-radiative decay pathway is restricted, leading to an increase in the fluorescence intensity and fluorescence lifetime. BODIPY 1c has been calibrated previously by the lab that designed it (Sherin et al., 2017), but we characterized the 1c that we synthesized (by Nanosyn) for our lab under our experimental conditions and with our imaging systems.

To perform quantitative measurement of viscosity, the lifetime of BODIPY 1c was calibrated as a function of viscosity using glycerol (Acros Organics, CAS 56-81-5) and methanol (Fisher Chemical, A456-1) mixtures varying from 30:70 to 100:0 (**Figure S1A-C**). BODIPY 1c was added to the 2 ml glycerol/methanol mixture at 1:500 rotor:mixture ratio and kept on a rotating vertical shaker (Labquake) at room temperature, away from the light, overnight for efficient mixing. For fluorescence lifetime imaging, the glycerol/methanol mixture was transferred to a glass well plate and imaged using Leica SP8­ FALCON system at 20 °C. Viscosity measurements of the above glycerol/methanol mixtures (20/80 to 100/0) were made with ARES-G2 rheometer (TA Instruments) with APS temperature control system and a 40 mm cone plate geometry. Samples for viscosity measurements were 0.8 ml and measured at shear rate from 10 to 100 1/s for 30s; the flow cycles were run thrice for each mixture. The plate unit was kept at a constant temperature of 20 °C with water circulating from a temperature-controlled water bath. A logarithmic plot of the fluorescence lifetime versus the mixture viscosity yielded a straight line (**Figure S1C**, r^2^ = 0.98) that obeys the Förster Hoffman equation (Förster & Hoffmann, 1971). This plot was very similar to that previously generated (Sherin et al., 2017) and served as a calibration graph to convert fluorescence lifetime to viscosity. We report our data as lifetimes because we cannot calibrate our sensor in the native hair bundle environment where cytoskeletal interactions and membrane proteins might alter the absolute value of viscosity. Our data does suggest that reporting an ‘effective viscosity’ is valid and discussing changes observed with the sensor as impacting viscosity is valid.

Artificial lipid vesicles were used as a control to validate dye properties. Liposomes were prepared with either 100% 1,2-dioleoyl-sn-glycero-3-phosphocholine (DOPC, Avanti Polar Lipids) or 70% Egg Sphingomyelin (Egg SM, Avanti Polar Lipids, 860061).and 30% Cholesterol (Ovine Cholesterol, Avanti Polar lipids, 700000). For 100% DOPC vesicles, the solvent of a 0.1 ml aliquot of 10 mg/ml DOPC in chloroform was evaporated in the vacuum to leave a dried lipid film. To prepare SM/Chol vesicles, the solvent containing 70 µl of 10 mM SM and 30 µl of 10 mM of cholesterol in chloroform was dried in the vacuum. Dried lipids were hydrated in the buffer (0.25ml for 100% DOPC and 0.3 ml for SM/Chol) with 100 mM KCl and 10 mM HEPES at pH 7.4 for an hour. The lipid solution was then extruded (Mini Extruder, Avanti Polar Lipids) through a 200 nm polycarbonate membrane 11 times. BODIPY 1c was added to the lipid solution in 1:200 rotor:lipid ratio to prevent dye aggregation and kept at 4 °C, away from light. For imaging, the liposomes were mounted on a slide in 0.5% agarose gel (Fisher Scientific) to keep them mechanically stable. Vesicles were prepared on the day of the experiment. As shown previously (Sherin et al., 2017), the highly viscous SM:Chol vesicles showed slower fluorescence decay curves and higher lifetime than pure DOPC vesicles **(Figure S1D-F**), thus validating 1c. Importantly, BODIPY 1c showed similar lifetime values in these vesicles as previously reported at 20 °C (Sherin et al., 2017).


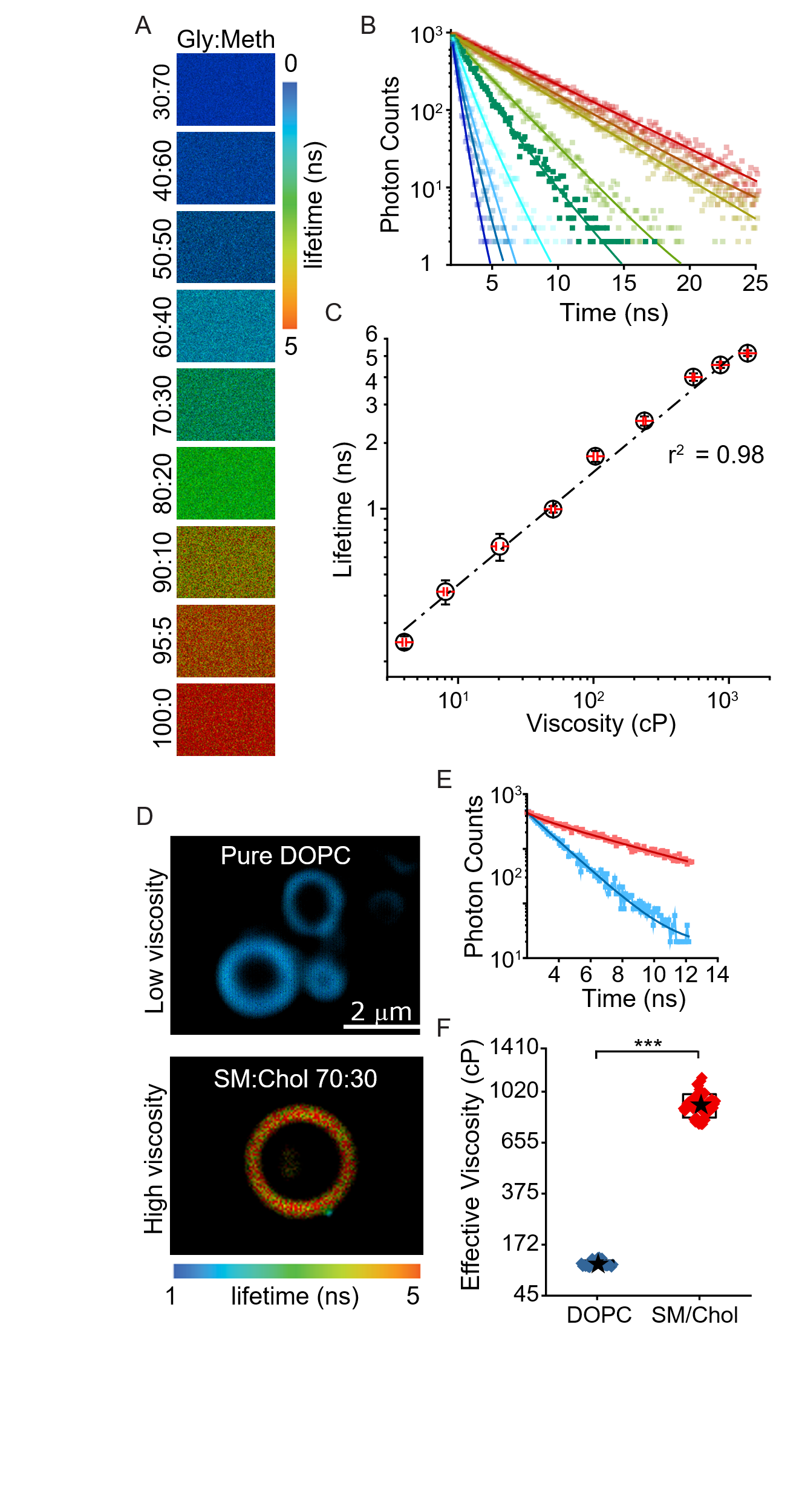


**Figure S1:** Validation and calibration of BODIPY 1c in model membranes and glycerol/methanol mixtures. A) FLIM images of BODIPY 1c in pure DOPC (top panel) and SM:Chol 70:30 (bottom panel) vesicles. B) Time-resolved fluorescence decays recorded and C) Lifetimes measured from pure DOPC (blue) and SM:Chol (red) vesicles. D) FLIM images and E) Time-resolved fluorescence decay of BODIPY 1c in glycerol/methanol mixtures of varying composition. F) Fluorescence lifetime vs. viscosity calibration obtained for BODIPY 1c in glycerol/methanol mixtures at 20 °C.

**Determining the BODIPY 1c concentration for cochlea**

BODIPY based molecular rotors are characterized by monoexponential fluorescence decays in homogeneous medium and in the absence of aggregates. Hence, the presence of biexponential decays can be either the presence of aggregates or lipid heterogeneities in the membrane. The aggregated species are characterized by a weak emission in the red region from 600-670 nm (Sherin et al., 2017). The aggregates cause quenching of the main emission band from 490-560 nm which renders the lifetime-viscosity calibration curve unstable. We, therefore, compared the decay curves recorded at 490-560 nm and 600-670 nm at a range of BODIPY 1c concentrations in cochlear hair bundles (**Figure S2A**) to determine an optimal incubation concentration for cochlear cells that is low enough to avoid dye aggregation and high enough to achieve good staining. If the decay curves from the two wavelength ranges are different, that indicates the presence of dye aggregation. The decay curves recorded from the two spectra for BODIPY 1c in cochlear hair bundles show evidence of dye aggregation at 16 and 12 mM but not at 10 mM (**Figure S2A**). Hence, we used a concentration of 8-10 mM for all our subsequent experiments. We also confirmed that the fluorescence lifetime measurements were independent of the dye concentration below 10 mM (**Figure S2B-D**).


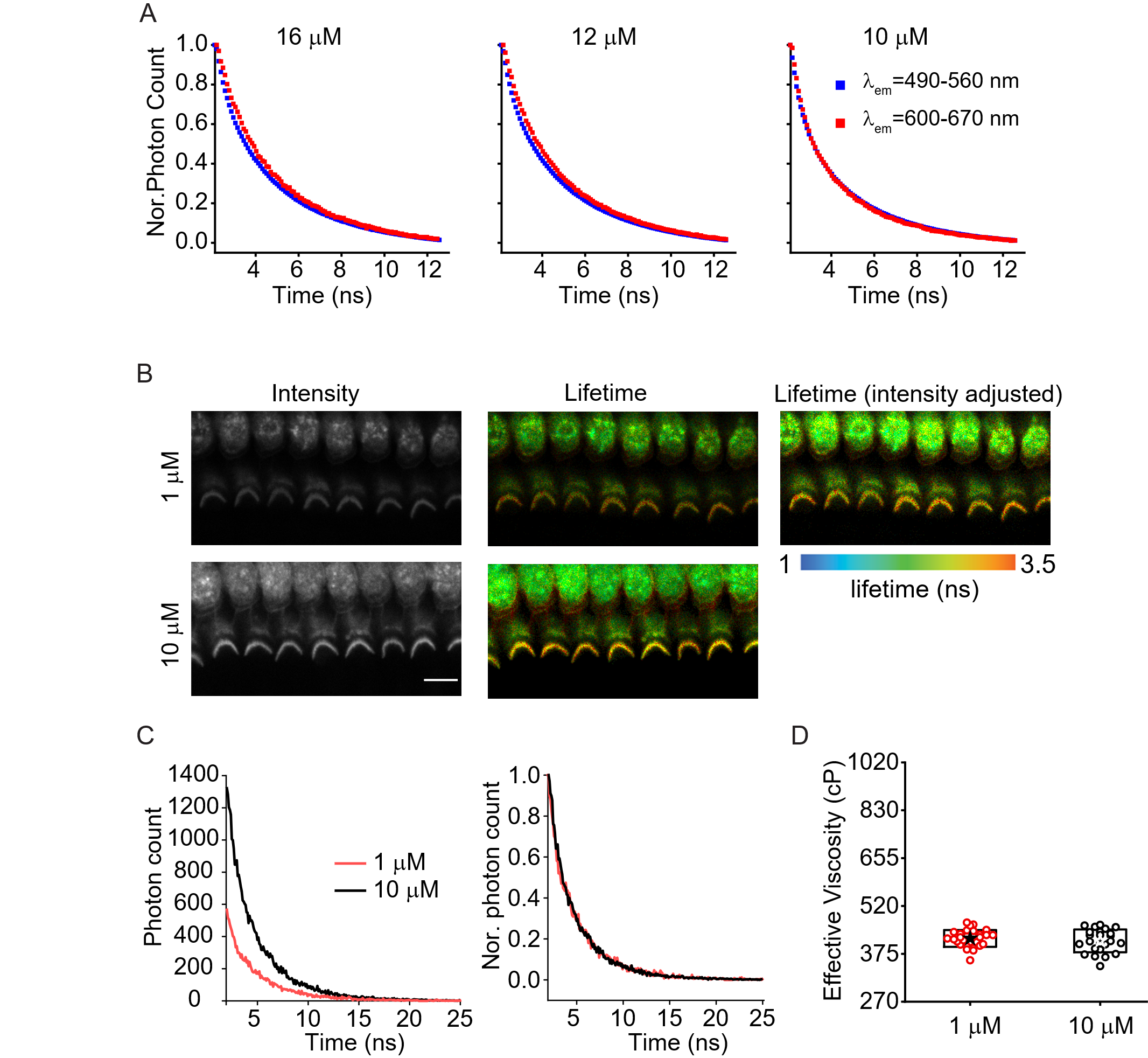


**Figure S2:** A) Time-resolved fluorescence decays recorded from P10 rat mid-apical cochlear hair bundles stained with different concentrations of BODIPY 1c (16, 12 and 10 μM) in PBS following excitation at 480 nm and detection in two spectral ranges 490-560 nm (monomers) and 600-670 nm (aggregates). B) Intensity and FLIM images, C) Time-resolved fluorescence decay curves and D) Measure lifetime of P10 rat mid-apical cochlear hair bundles at 1 mM (top row in B, red lines and symbols) and 10 mM (bottom row in B, black lines and symbols) concentrations of BODIPY 1c. Scale bar = 10 µm.
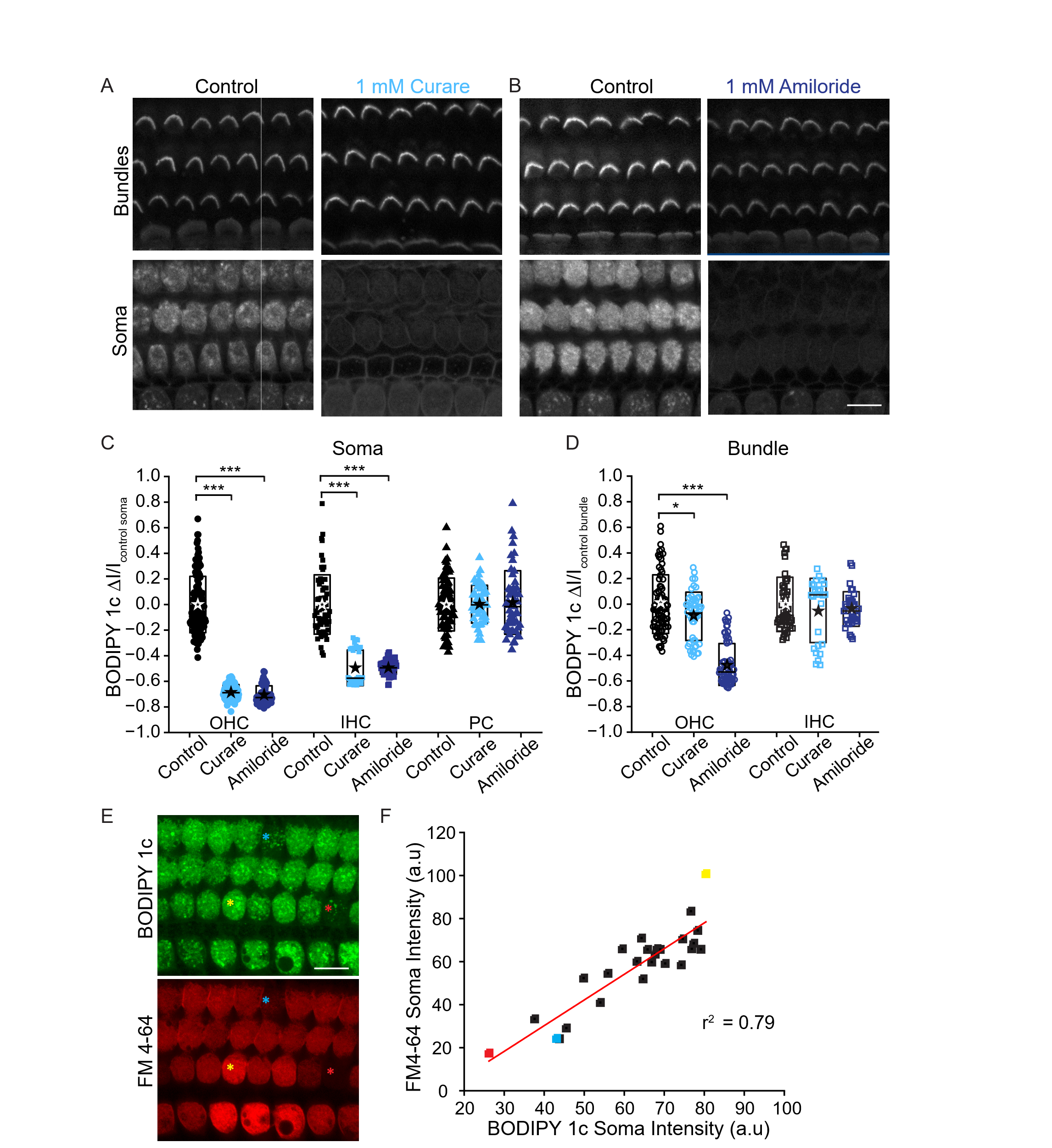


**Figure S3:** A, B) Intensity images of BODIPY 1c in P10 rat mid-apical turn of control, 1mM curare and 1mM amiloride treated organ of Corti with focus plane at the hair bundles (top rows) and soma (bottom panels). C, D) Quantification of BODIPY 1c intensity in the C) soma of the hair cells and D) the hair bundles. E) Intensity images of BODIPY 1c (top panel) and FM 4-64 (bottom panel) from the soma of same sample (P10 rat mid-apical turn). Asterisks of different colors are used to highlight a particular cell. F) Plot showing the correlation between the soma intensity of BODIPY 1c and FM 4-64. Boxes in C and D represent the SD, and the star symbol indicates the mean. Each data point corresponds to a hair bundle or a cell (for soma). **p* < 0.05, ***p* < 0.01, ****p* < 0.001. Scale bar = 10 µm.


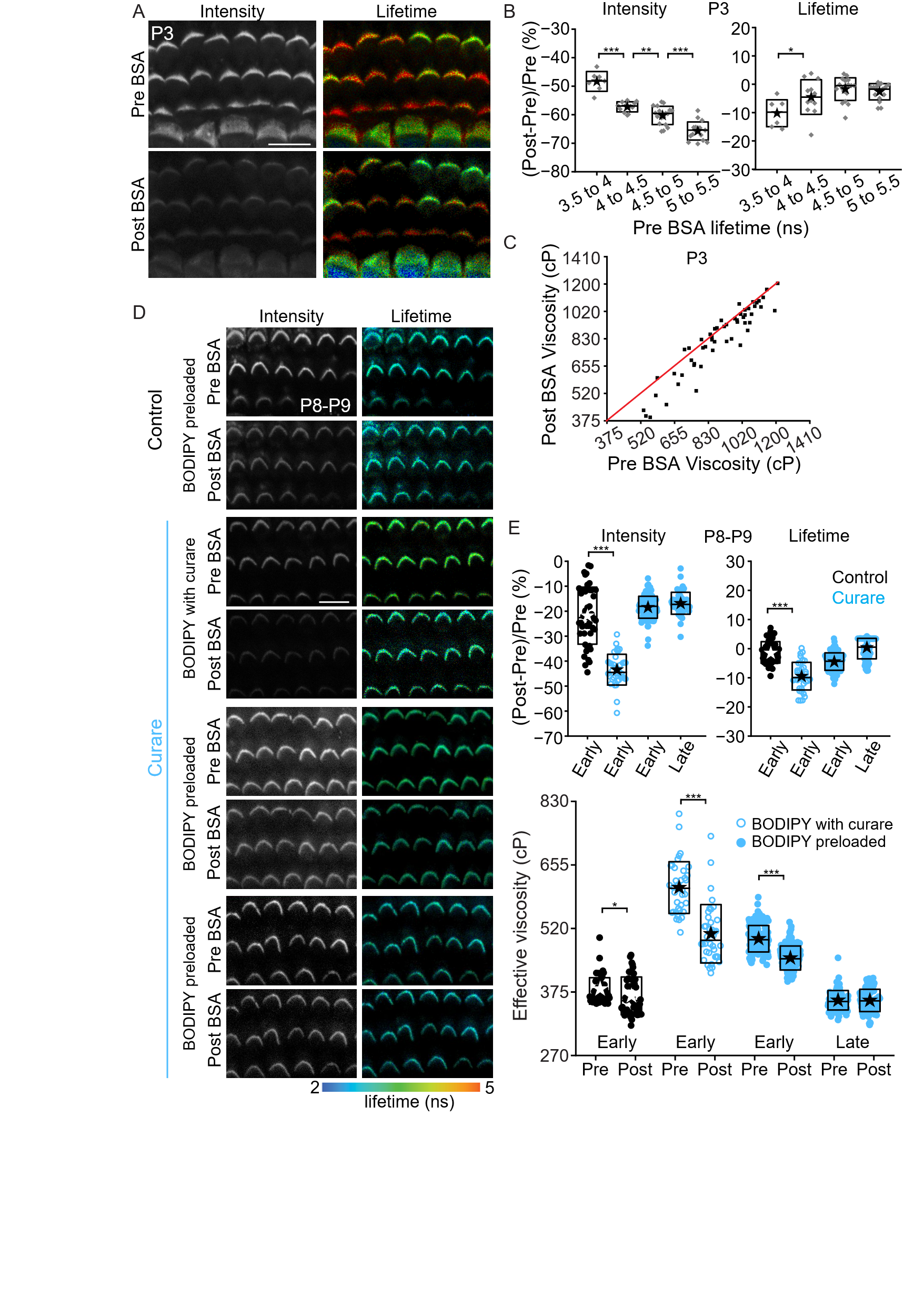


**Figure S4:** A) Example intensity and lifetime images of the hair bundles pre (top) and post (bottom) BSA back extraction of BODIPY 1c from a P3 rat mid-apical turn. B) Quantification of the percentage change in the intensity and lifetime with BSA back extraction plotted against the pre-BSA lifetime of the hair bundle. C) Plotting the effective viscosity measurements before and after BSA back extraction. D) Example intensity and lifetime images of the P8-P9 OHBs in control and curare treated (BODIPY 1c is either preloaded or applied with curare) conditions. E) Quantifications of the percentage change in the intensity and lifetime and the effective viscosity measured pre and post BSA back extraction with different conditions stated in D. **p* < 0.05, ***p* < 0.01, ****p* < 0.001. Scale bar = 10 µm.


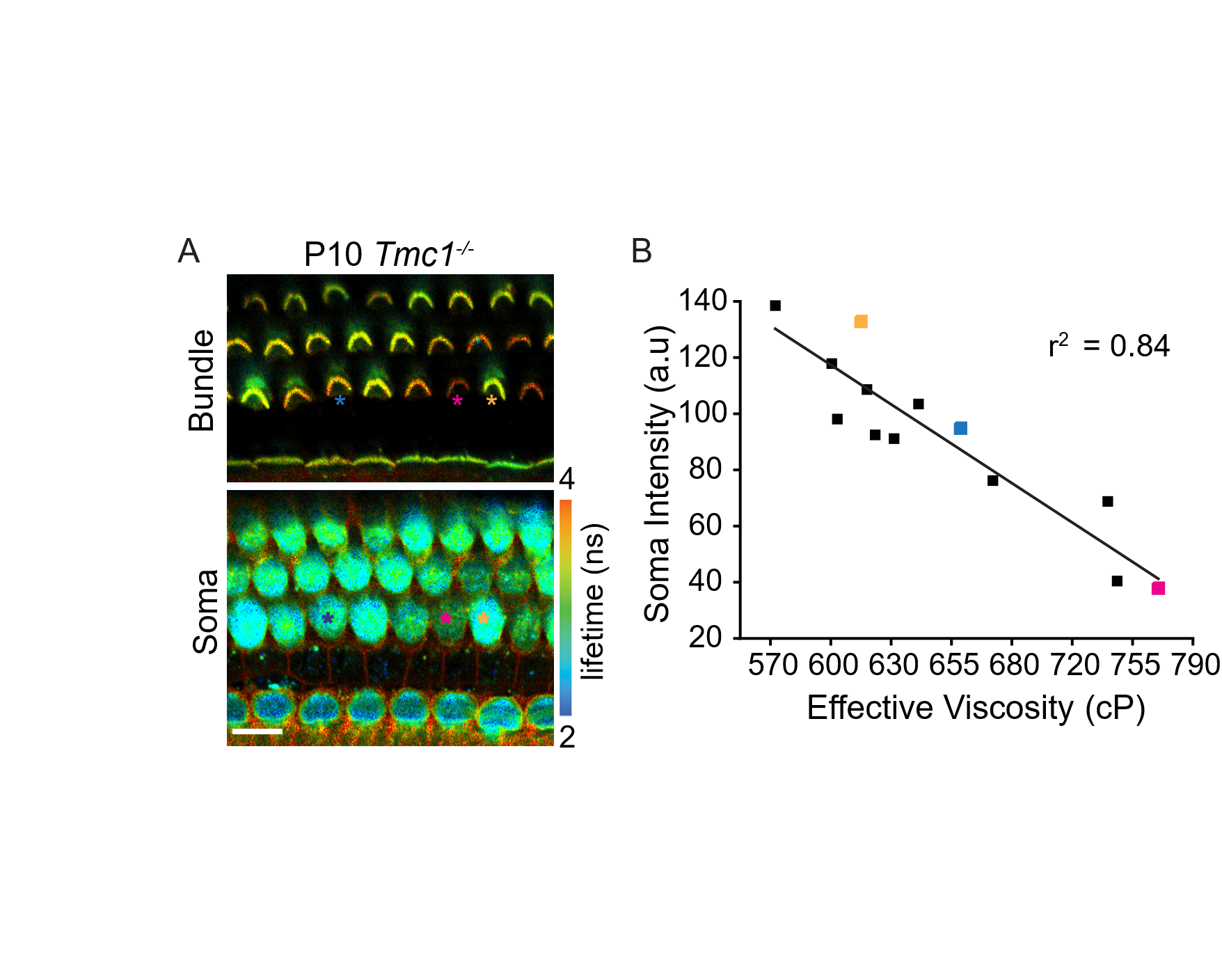


**Figure S5:** A) An example FLIM image of the hair bundles (top) and the corresponding soma (bottom) from a P10 TMC1 KO mid-apical turn showing the range of lifetimes and the soma intensity seen from the neighboring HCs. B) The bundle viscosity and the soma intensity of the corresponding HC was measured for the images shown in A) and plotted to show a strong correlation between both (r^2^= 0.84, *p* < 0.001). Scale bar = 10 µm.


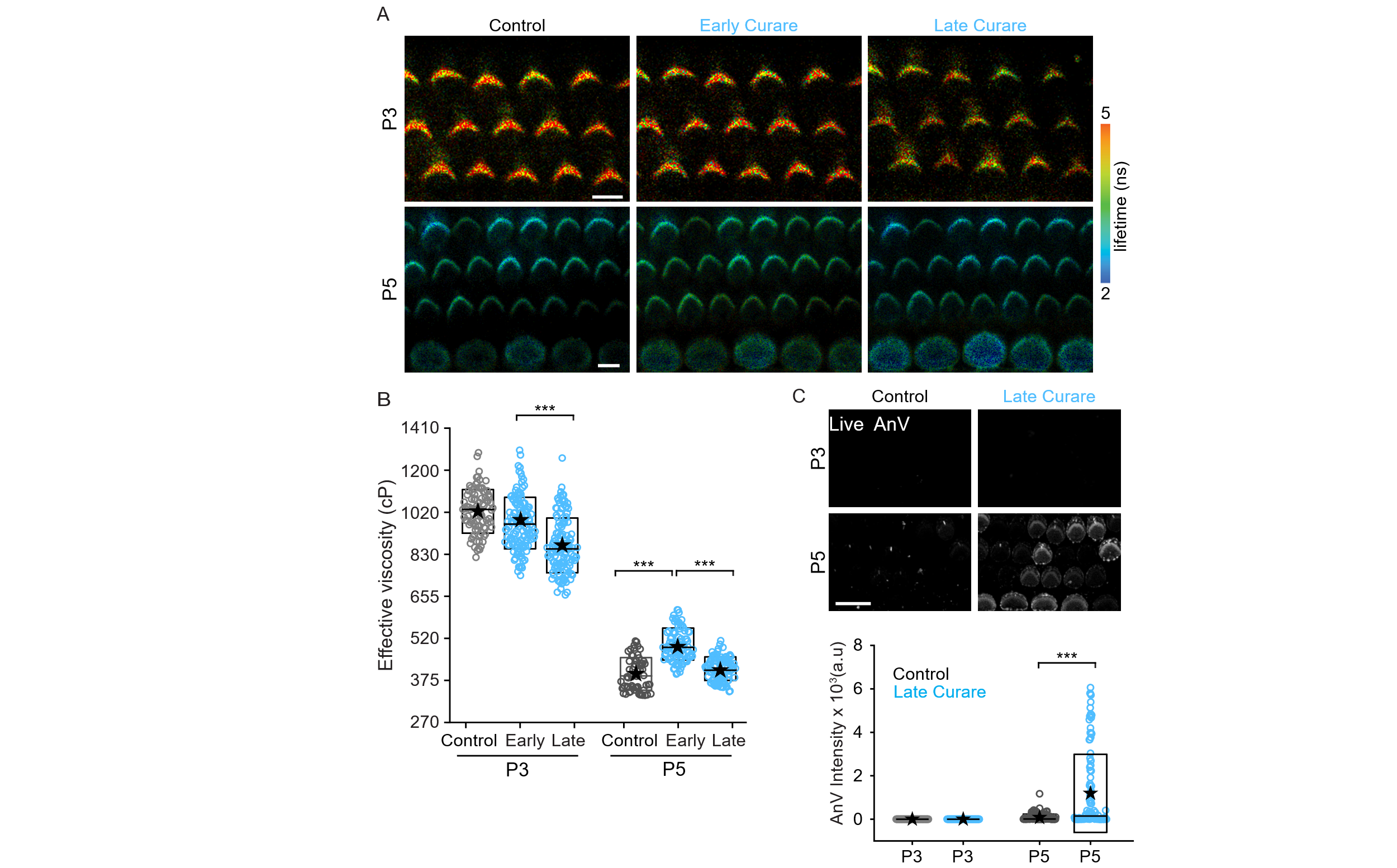


**Figure S6:** A) FLIM images of OHCs from P3 and P5 rat mid-apical turns untreated (control) and treated with 1mM curare for ~10 mins (early) and 30 mins (late). B) Quantification of lifetime for OHC bundles treated as in A (n=3 animals in each group). C) Live confocal images of OHCs and IHCs labelled with AnV from control and late curare treated and the correspsonding quantification. Boxes in B and C represent the SD, and the star symbol indicates the mean. Each data point corresponds to a hair bundle ****p* < 0.001. Scale bar = 5 µm in A and 10 µm in C.


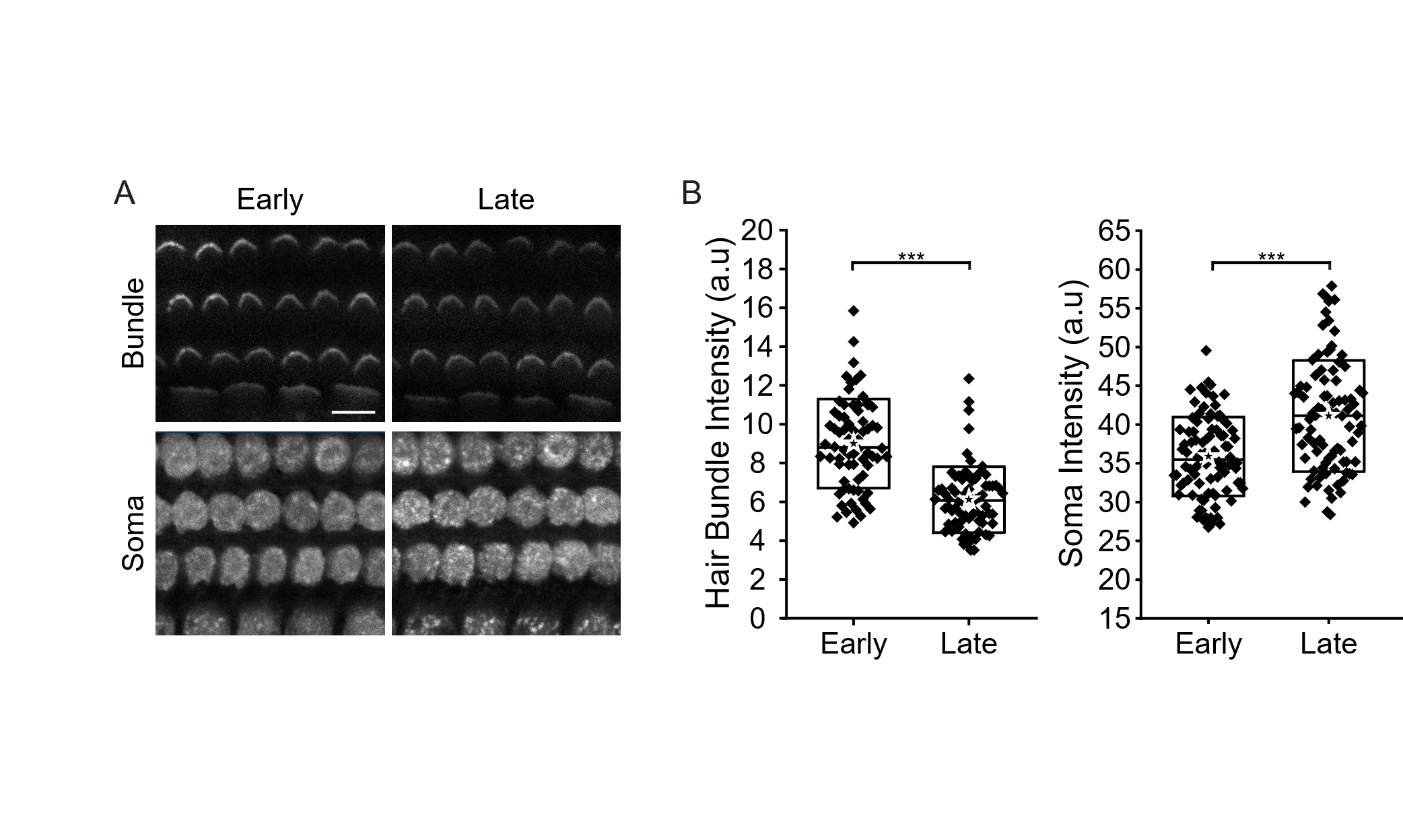


**Figure S7:** A) Intensity images of BODIPY 1c at the hair bundle and soma from a P9 rat mid-apical turn in control condition at early (10 mins) and late (30 mins) time points. B) Quantification of BODIPY 1c intensity in the hair bundle and the soma of hair cells at the differnet time points . Boxes in B represent the SD, and the star symbol indicates the mean. Each data point corresponds to a hair bundle or a cell (for soma).****p* < 0.001. Scale bar = 10 µm.


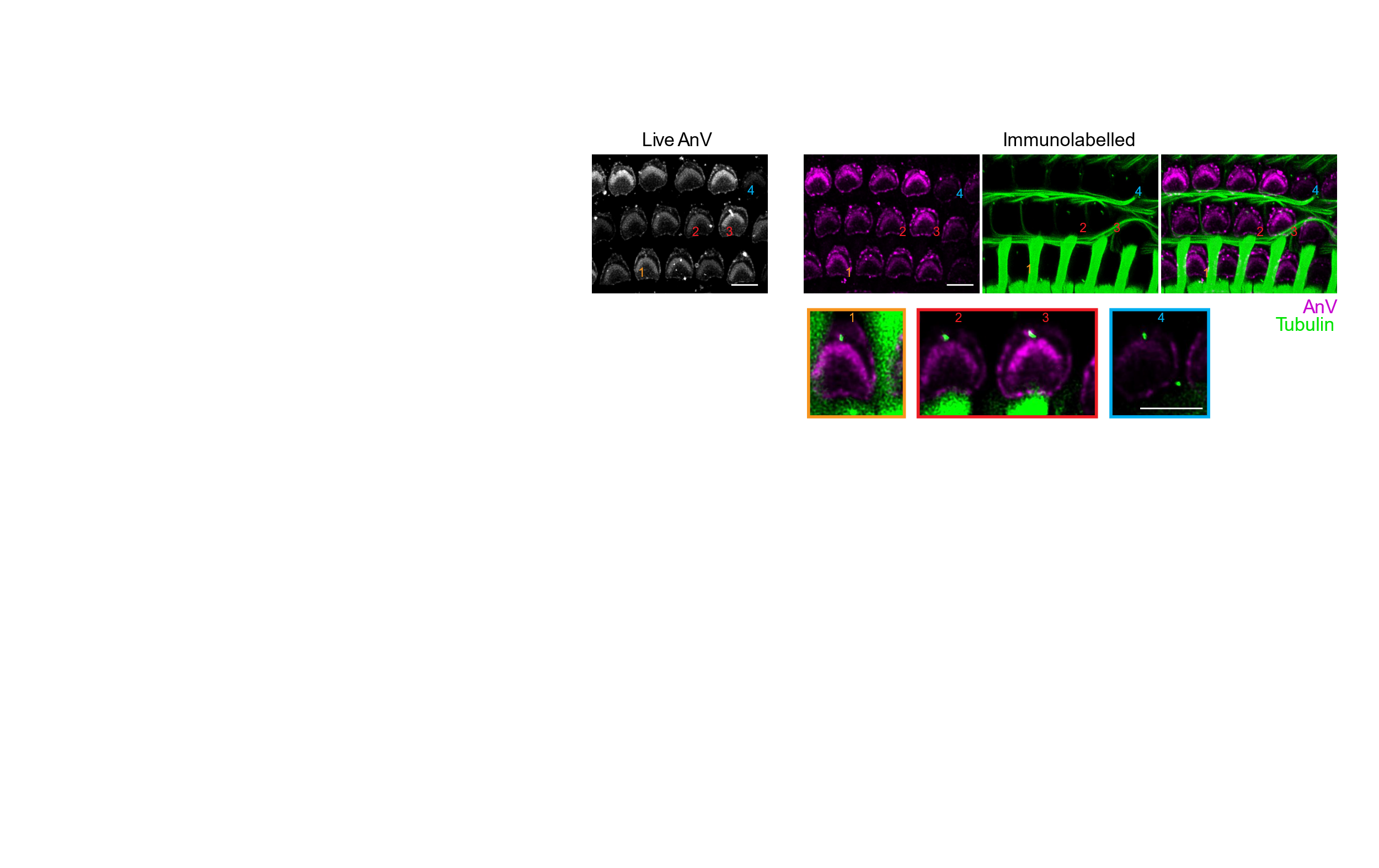


**Figure S8:** Confocal images of a P9 rat organ of Corti with hair bundles labelled with AnV and with a acetyl-tubulin antibody for kinocilium.Specific tubulin staining is detected in the hair bundles highlighted with numbers 1-4. Scale bars = 5 μm.

**References**

R., Krams, R., Bull, J. A., Brooks, N. J., & Kuimova, M. K. (2015). Imaging phase separation in model lipid membranes through the use of BODIPY based molecular rotors. *Physical Chemistry Chemical Physics*, *17*(28), 18393-18402. <https://doi.org/10.1039/c5cp01937k>

Förster, T., & Hoffmann, G. (1971). Die Viskositätsabhängigkeit der Fluoreszenzquantenausbeuten einiger Farbstoffsysteme. *Zeitschrift für Physikalische Chemie*, *75*(1_2), 63-76. <https://doi.org/doi:10.1524/zpch.1971.75.1_2.063>

Kuimova, M. K., Yahioglu, G., Levitt, J. A., & Suhling, K. (2008). Molecular rotor measures viscosity of live cells via fluorescence lifetime imaging. *Journal of the American Chemical Society*, *130*(21), 6672-+. <https://doi.org/10.1021/ja800570d>

López-Duarte, I., Vu, T. T., Izquierdo, M. A., Bull, J. A., & Kuimova, M. K. (2014). A molecular rotor for measuring viscosity in plasma membranes of live cells. *Chemical Communications*, *50*(40), 5282-5284. <https://doi.org/10.1039/c3cc47530a>

Sherin, P. S., Lopez-Duarte, I., Dent, M. R., Kubankova, M., Vysniauskas, A., Bull, J. A., Reshetnikova, E. S., Klymchenko, A. S., Tsentalovich, Y. P., & Kuimova, M. K. (2017). Visualising the membrane viscosity of porcine eye lens cells using molecular rotors. *Chem Sci*, *8*(5), 3523-3528. <https://doi.org/10.1039/c6sc05369f>
